# Multicenter Development and Prospective Validation of eCARTv5: A Gradient-Boosted Machine-Learning Early Warning Score

**DOI:** 10.1097/CCE.0000000000001232

**Published:** 2025-03-26

**Authors:** Matthew M. Churpek, Kyle A. Carey, Ashley Snyder, Christopher J Winslow, Emily Gilbert, Nirav S Shah, Brian W. Patterson, Majid Afshar, Alan Weiss, Devendra N. Amin, Deborah J. Rhodes, Dana P. Edelson

**Affiliations:** 1 Department of Medicine, University of Wisconsin-Madison, Madison, WI.; 2 Department of Biostatistics and Medical Informatics, University of Wisconsin-Madison, Madison, WI.; 3 Department of Medicine, University of Chicago, Chicago, IL.; 4 AgileMD, San Francisco, CA.; 5 Department of Medicine, Endeavor Health, Evanston, IL.; 6 Department of Medicine, Loyola University Medical Center, Chicago, IL.; 7 Department of Emergency Medicine, University of Wisconsin-Madison, Madison WI.; 8 BayCare, Clearwater, FL.; 9 Department of Medicine, Yale University, New Haven, CT.

**Keywords:** artificial Intelligence, clinical deterioration, early warning score, machine learning, rapid response systems

## Abstract

**BACKGROUND:**

Early detection of clinical deterioration using machine-learning early warning scores may improve outcomes. However, most implemented scores were developed using logistic regression, only underwent retrospective validation, and were not tested in important subgroups.

**OBJECTIVE:**

The objective of our multicenter retrospective and prospective observational study was to develop and prospectively validate a gradient-boosted machine model (eCARTv5) for identifying clinical deterioration on the wards.

**DERIVATION COHORT:**

All adult patients admitted to the inpatient medical-surgical wards at seven hospitals in three health systems for model development (2006–2022).

**VALIDATION COHORT:**

All adult patients admitted to the inpatient medical-surgical wards and at 21 hospitals from three health systems for retrospective (2009–2023) and prospective (2023–2024) external validation.

**PREDICTION MODEL:**

Predictor variables (demographics, vital signs, documentation, and laboratory values) were used in a gradient-boosted trees algorithm to predict ICU transfer or death in the next 24 hours. The developed model (eCARTv5) was compared with the Modified Early Warning Score (MEWS), the National Early Warning Score (NEWS), and eCARTv2 using the area under the receiver operating characteristic curve (AUROC).

**RESULTS:**

The development cohort included 901,491 admissions, the retrospective validation cohort included 1,769,461 admissions, and the prospective validation cohort included 205,946 admissions. In retrospective validation, eCARTv5 had the highest AUROC (0.834; 95% CI, 0.834–0.835), followed by eCARTv2 (0.775 [95% CI, 0.775–0.776]), NEWS (0.766 [95% CI, 0.766–0.767]), and MEWS (0.704 [95% CI, 0.703–0.704]). eCARTv5’s performance remained high (AUROC ≥0.80) across a range of patient demographics, clinical conditions, and during prospective validation.

**CONCLUSION:**

We developed eCARTv5, which performed better than eCARTv2, NEWS, and MEWS retrospectively, prospectively, and across a range of subgroups. These results served as the foundation for Food and Drug Administration clearance for its use in identifying deterioration in hospitalized ward patients.

**KEY POINTS**

**Question:** Can a more advanced machine learning model identify deterioration in hospitalized ward patients more accurately than previously published tools?

**Findings:** In retrospective validation, a gradient-boosted machine model (eCARTv5) had the highest area under the receiver operating characteristic curve when compared with the Modified Early Warning Score, the National Early Warning Score, and eCARTv2. eCARTv5’s performance remained high across a range of patient demographics, clinical conditions, and during prospective validation.

**Meaning:** This article evaluating eCARTv5’s performance served as the foundation for Food and Drug Administration clearance for identifying deterioration in hospitalized ward patients.

Clinical deterioration occurs in up to 5% of hospitalized patients, and early identification is associated with improved outcomes (1–9). These events are often heralded by deranged physiology, leading to the development of early warning scores aimed at identifying high-risk patients (10, 11). Early warning scores have evolved from aggregated weighted scores, such as the Modified Early Warning Score (MEWS) (12), which can be calculated by hand, to those based on logistic regression (1, 2), which can be summed with a calculator or spreadsheet, and more recently, to advanced machine learning models, such as gradient boosted machines (GBM), which can be more accurate in large datasets (13–16).

While machine-learning models may decrease false alarms and increase detection rates, they can suffer from poor performance when trained on insufficiently sized or representative datasets (17, 18). Furthermore, their use in clinical practice raises concerns about model fairness and bias, and little is known regarding how they perform across a range of important patient subgroups. Evaluating models across subgroups, such as age, sex, and race, is critical to ensuring the fairness of these tools in practice so that they have the potential to benefit all patients. Finally, most prior work has been done retrospectively (11), and it is not known whether these complex models will perform similarly in prospective production environments.

Therefore, we aimed to develop and externally validate a GBM model for identifying clinical deterioration in a large, geographically diverse set of hospitals. After retrospective validation, which included extensive subgroup analyses, we tested performance prospectively. These results served as the foundation for 510(k) clearance by the U.S. Food and Drug Administration (FDA) for inpatient clinical deterioration detection outside the ICU (ICU) (19).

## METHODS

### Study Setting and Population

Hospitalized adults (18 years old or older) admitted to the medical-surgical wards were eligible for inclusion in this observational cohort study. Patients who were only admitted to the ICU, labor and delivery, or emergency department and were never transferred to a medical-surgical (non-ICU) unit during their hospital encounter were excluded. The model, eCARTv5, was developed in a dataset of seven hospitals from three health systems in Illinois (D1–D3), spanning 2006 to 2022. The model was then externally validated in two phases: (1) a retrospective cohort of admissions to 21 hospitals from three health systems (R1–R3) in Florida, Wisconsin, Connecticut, and Rhode Island (2009–2023); and (2) a prospective cohort of consecutive admissions to the same hospitals (P1–P3). See **Table E1** (http://links.lww.com/CCX/B484) for a list of included hospitals and years of data included in the study. eCARTv5 was compared with the MEWS (12) and the National Early Warning Score (NEWS) (20), commonly used tools for clinical deterioration. The original NEWS was chosen as a comparator over NEWS2 because choosing the Spo_2_ scale in NEWS2 requires real-time clinician determination (21). The first clinically implemented eCART version (eCARTv2), which is based on logistic regression, was also included as a comparator (1). The study was approved by the following Institutional Review Boards (IRBs): University of Chicago Biological Sciences Division IRB (#18-0447), Loyola University Chicago Health Sciences Division IRB (#215437), NorthShore University HealthSystem Research Institute IRB (#EH16-210T), University of Wisconsin-Madison Minimal Risk Research IRB (#2019-1258), BayCare Health System IRB (#2022.014-B.MPH; 2022.015-B.MPH), and Yale Human Research Protection Program IRBs (#2000035317). Each IRB waived study-specific informed consent. Procedures followed the ethical standards of the responsible institutional committee on human experimentation and the Helsinki Declaration of 1975.

### Main Outcome Measure

The study outcome was clinical deterioration, defined as a ward death or ICU transfer from the wards within 24 hours of a score (1, 13, 20). Death was determined using the discharge disposition in the electronic health record (EHR), with the time of death being the last recorded vital sign. ICU transfer was defined as a direct ward-to-ICU transfer and was determined using the transfer disposition in the EHR.

### Predictor Variables

Ninety-seven features were included as predictor variables in the eCARTv5 model. These variables included patient characteristics (e.g., age, body mass index), vital signs, laboratory values, time of day, and nursing/respiratory therapist documentation (e.g., delivered oxygen percentage, Braden scale), as well as trends (22). A full list of model predictor variables is found in **Table E2** (http://links.lww.com/CCX/B484).

Nonphysiologic flowsheet data were considered input errors and treated as missing, as per prior publications (**Table E3**, http://links.lww.com/CCX/B484). If no prior values were available at a specific time, then the most recent prior value, if available, was pulled forward. If no prior values were available, the variable was left as missing. Given the dynamic nature of blood gas and lactate values and the tendency of providers to only order them on actively deteriorating patients, those values were only pulled forward for 24 hours, after which they were treated as missing.

### Model Development

A GBM model was developed to predict clinical deterioration in the training data using discrete-time survival analysis (1, 13, 23). Time was discretized into 8-hour blocks, and the data at the beginning of each segment were used to predict whether an outcome occurred within the following 8 hours. This approach allowed the inclusion of time-varying predictors, removed the bias of sicker patients receiving more frequent measurements, and provided results analogous to a Cox survival model (23). Because tree-based models can perform poorly in imbalanced data (i.e., when the outcome of interest is uncommon), down-sampling of the training dataset to obtain a 50% outcome prevalence was performed before model fitting (13). Model hyperparameters were tuned in the training cohort using five-fold cross-validation to maximize the area under the receiver operating characteristic curve (AUROC). No variable selection was performed, as research has shown that this may degrade the accuracy of tree-based algorithms (24). Variable importance was calculated based on the improvement of each split averaged across all trees (17). Additional details can be found in the **Online Data Supplement** (http://links.lww.com/CCX/B484).

### Retrospective and Prospective Score Calculation

For model validation, data pre-processing was performed in the same manner as during model development, except that in the validation cohorts, data were not blocked, and no down-sampling was performed. Specifically, each time a new observation was recorded in the EHR, predicted probabilities from eCARTv5, eCARTv2, MEWS, and NEWS values were calculated. The transformed eCARTv5 model output probabilities were then scaled to eCARTv5 scores ranging from 0 to 100 for ease of interpretation. For the retrospective validation, these scores were calculated on a static multicenter dataset stored on secured laboratory servers. In the prospective validation, the model features, eCARTv5 scores, and outcomes were collected in real-time using Health Level-Seven Version 2 (HL-7 V2) messaging standard interfaces, a clinical data standard to protocolize how data are shared and exchanged in EHR operations, and stored on cloud-based computing and storage resources hosted at Amazon Web Services. The engine calculated scores as data became available, with the first score filed back to the EHR for any given time. For the prospective cohort, eCARTv2, MEWS, and NEWS were not calculated in real time. In a subset of encounters from the prospective cohort, retrospective eCARTv5 scores were also calculated to directly compare the impact of the different methodologies on scoring.

### Statistical Analysis

Descriptive statistics were used to characterize patient demographics across the separate development, retrospective validation, and prospective validation cohorts. Model performance was calculated by assessing the ability of the scores at each observation time to predict clinical deterioration in the following 24 hours. Discrimination was measured using the AUROC and then compared using DeLong’s method (25). To demonstrate that the inclusion of multiple observations per encounter did not bias the results, a 100-iteration cluster bootstrapped analysis with replacement was run in the retrospective and prospective cohorts (26, 27). Subgroup analyses were performed in the retrospective validation cohort across patient demographics and clinical conditions (see **Supplemental Methods**, http://links.lww.com/CCX/B484, for definitions). Sensitivity, specificity, and positive and negative predictive values were calculated for each threshold, with CIs calculated using the Clopper-Pearson method. Performance at a moderate-risk and high-risk threshold for each score (eCARTv5 of ≥93 and ≥97; eCARTv2 of ≥21 and ≥35; NEWS ≥5 and ≥7; MEWS ≥3 and ≥4) was also compared. Model calibration was assessed in the prospective validation by comparing observed to expected deterioration rates across eCARTv5 score values. Analyses were performed using Stata version 16.1 (StataCorps; College Station, TX) and R version 4.2.1 (The R Foundation for Statistical Computing, Vienna, Austria).

## RESULTS

The training dataset included 901,491 adult inpatient admissions with ward stays at seven hospitals from three health systems. The validation cohorts included 1,769,461 retrospective admissions and 205,946 nonoverlapping prospective admissions to 21 hospitals from three health systems. In the retrospective cohort, there was a median of 46 observations per encounter (interquartile range [IQR], 24–89), while in the prospective cohort, there was a median of 74 observations per encounter (IQR, 42–128). Patient demographics varied across the three cohorts (**Table 1**) and the different geographic regions (**Table E4**, http://links.lww.com/CCX/B484). When compared with the two validation cohorts, the training cohort had a higher proportion of black patients (32% vs 14%) and a lower percentage of sepsis, COVID-19, heart failure, and chronic obstructive pulmonary disease (COPD). Meanwhile, the prospective validation cohort differed from the retrospective validation cohort in having a higher median age (66 vs. 62 yr), a shorter length of stay (65 vs. 72 hr), and fewer encounters with surgical procedures. There was also variation in missing variables across the health systems (**Tables E5** and **E6,** http://links.lww.com/CCX/B484). Most notably, R3 had higher rates of missing Braden scores and respiratory rate trends, suggesting a lower frequency of respiratory rate documentation, which was more pronounced in the prospective cohort (P3). R1 had higher rates of missing hematology laboratories, particularly WBC differential distributions. R2 had higher missing rates for mental status.

**TABLE 1.** Patient Characteristics By Cohort

Patient Characteristics	Derivation Cohort	Retrospective Validation Cohort	Prospective Validation Cohort
Hospitals, *N*	6	21	21
Encounters, *N*	901,491	1,769,461	205,946
Admission age, yr, median (IQR)	61 (44–74)	62 (45–75)	66 (52–78)
Female sex	512,198 (56.8%)	993,297 (56.1%)	113,422 (55.1%)
Race: American Indian or Alaska Native	1,445 (0.2%)	6,468 (0.4%)	538 (0.3%)
Race: Asian/Mideast Indian	23,334 (2.6%)	26,681 (1.5%)	2,928 (1.4%)
Race: Black/African American	286,901 (31.8%)	252,982 (14.3%)	28,659 (13.9%)
Race: Pacific Islander/ Hawaiian Native	666 (0.1%)	2,496 (0.1%)	224 (0.1%)
Race: White/Caucasian	496,154 (55.0%)	1,384,075 (78.2%)	158,435 (76.9%)
Race: Other	92,991 (10.3%)	96,759 (5.5%)	15,162 (7.4%)
Surgical	312,153 (34.6%)	539,875 (30.5%)	49,265 (23.9%)
Obstetric	65,594 (7.3%)	154,759 (8.7%)	12,653 (6.1%)
Sepsis	240,651 (26.7%)	639,802 (36.2%)	77,296 (37.5%)
COVID-19	4,365 (0.5%)	49,834 (2.8%)	7,920 (3.8%)
Congestive heart failure	131,644 (14.6%)	306,140 (17.3%)	44,223 (21.5%)
Chronic pulmonary disease	150,043 (16.6%)	443,263 (25.1%)	53,820 (26.1%)
Length of stay, hr, median (IQR)	70 (37–124)	72 (43–130)	65 (35–116)
Ward to ICU transfer	32,320 (3.6%)	57,789 (3.3%)	6,143 (3.0%)
Mortality	10,568 (1.2%)	26,319 (1.5%)	2,373 (1.2%)

IQR = interquartile range.

**Figure 1.**
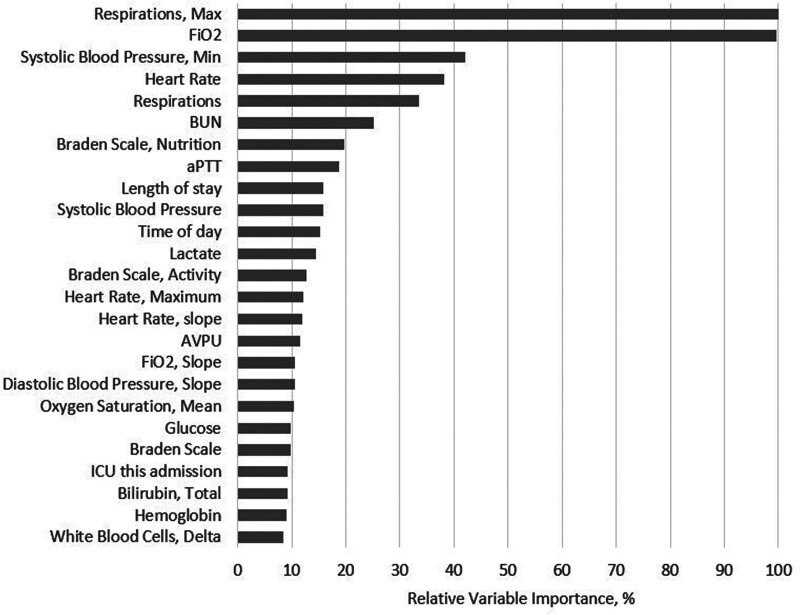
Variable importance plot illustrating the top 25 most important variables in the eCARTv5 model. aPTT = activated partial thromboplastin clotting time, AVPU = alert, responds to voice, responds to pain, unresponsive, BUN = blood urea nitrogen, Fio_2_ = fraction of inspired oxygen.

The most important variables in the final eCARTv5 model were maximum respiratory rate in the prior 24 hours, delivered Fio_2_, minimum systolic blood pressure in the prior 24 hours, and heart rate (**Fig. 1**). Partial plots illustrating the relationship between the values of these variables and the risk of deterioration are shown in **Figure E1** (http://links.lww.com/CCX/B484).

**Figure 2.**
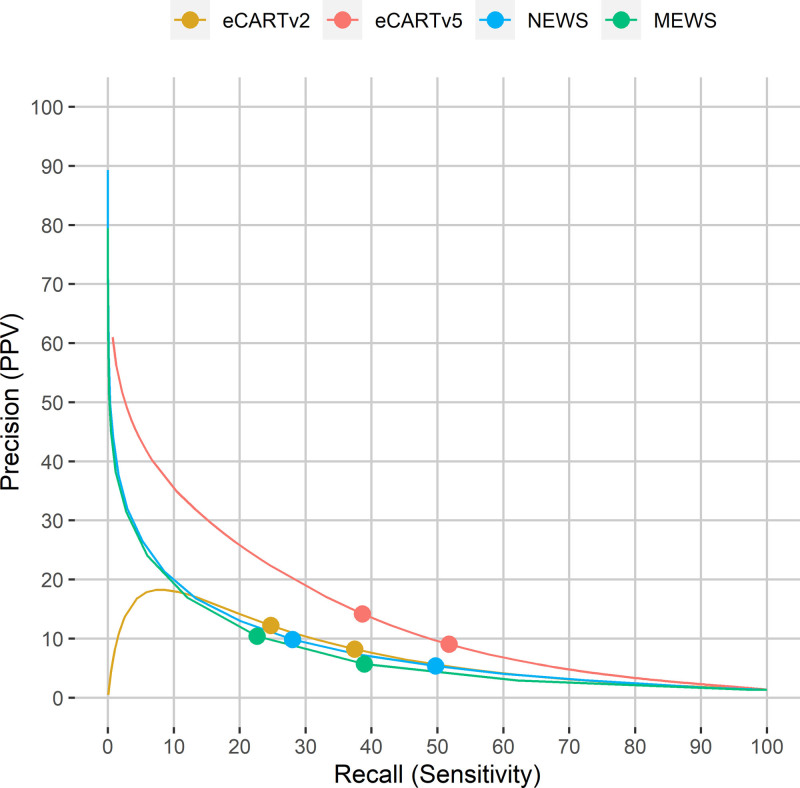
Precision-recall curves of the risk scores in the full retrospective dataset (*n* = 132,873,833 observations). Sensitivity is plotted along the *x*-axis and positive predictive value is plotted along the *y*-axis for eCARTv5, eCARTv2, National Early Warning Score (NEWS), and modified early warning score (MEWS). The markers on the lines correspond to a MEWS of 3 and 4, NEWS of 5 and 7, eCARTv2 of 21 and 35, and eCART of 93 and 97, representing commonly used moderate (higher sensitivity) and high-risk (higher positive predictive value [PPV]) thresholds for each score.

In the retrospective validation dataset, 132,873,833 eCARTv5, eCARTv2, MEWS, and NEWS were calculated. The AUROC for eCARTv5 was 0.834 (0.834, 0.835) for the full retrospective cohort (**Table 2**). eCARTv5 consistently outperformed eCARTv2 (AUROC, 0.775 [0.775–0.776]) and NEWS (AUROC, 0.766 [0.766–0.767]), which consistently outperformed MEWS (AUROC, 0.704 [0.703–0.704]). As shown in **Table E7** (http://links.lww.com/CCX/B484) eCARTv5 had the highest discrimination in every hospital included in the study. The bootstrapped analysis that accounted for multiple observations per patient demonstrated similar results to the main analysis, with eCARTv5 outperforming the other scores in both the retrospective and prospective cohorts (**Table E8**, http://links.lww.com/CCX/B484). eCARTv5’s sensitivity in the retrospective cohort at the moderate-risk threshold (≥93) was 51.8%, with a positive predictive value (PPV) of 9.0%. At the high-risk threshold (≥97), PPV increased to 14.2% with a decrease in sensitivity to 38.6% (**Table E9**, http://links.lww.com/CCX/B484). eCARTv2 had a 37.4% sensitivity and 8.2% PPV at the moderate-risk threshold (≥21) compared with a 24.7% sensitivity and 12.2% PPV at the high-risk threshold (≥35) (**Table E10**, http://links.lww.com/CCX/B484). In contrast, MEWS had sensitivities of 38.9% and 22.6% at the moderate (≥3) and high-risk (≥4) thresholds, with corresponding PPVs of 5.7% and 10.4%, respectively, while NEWS had sensitivities of 49.7% and 28.0% at the moderate (≥5) and high-risk (≥7) thresholds, with corresponding PPVs of 5.4% and 9.9%, respectively (**Tables E11** and **E12**, http://links.lww.com/CCX/B484). The precision-recall curve, **Figure 2**, plots PPV as a function of sensitivity and demonstrates a consistently more favorable tradeoff between sensitivity and PPV for eCARTv5 compared with eCARTv2, NEWS, and MEWS. At the moderate-risk threshold, NEWS provided the longest lead time before the deterioration event (17 hr [IQR, 1–73]), followed by eCARTv5 (16 hr [IQR, 1–68]), MEWS (13 hr [IQR, 1–66]), and then eCARTv2 (9 hr [IQR, 0–57]). At the high-risk threshold, eCARTv5 alerted a median of 5 (IQR, 0–43) hours in advance of clinical deterioration, significantly earlier than NEWS (3 hr (IQR, 0–40), MEWS (2 hr [IQR, 0–30]), and eCARTv2 (2 hr [IQR, 0–30]) (*p* < 0.01 for all comparisons).

**TABLE 2.** Area Under the Receiver Operating Characteristic Curve (AUROC) of the Risk Scores for Predicting the Outcome of Clinical Deterioration Within 24 hr in the External Retrospective and Prospective Validation Cohorts

Cohort	Encounters, *n*	Observations, *n*	eCARTv5 AUROC (95% CI)	eCARTv2 AUROC (95% CI)	National Early Warning Score AUROC (95% CI)	Modified Early Warning Score AUROC (95% CI)
Retrospective (all)	1,769,461	132,873,833	0.834 (0.834–0.835)	0.775 (0.775–0.776)	0.766 (0.766–0.767)	0.704 (0.703–0.704)
Retrospective (R1)	246,949	19,262,093	0.861 (0.861–0.862)	0.789 (0.788–0.790)	0.775 (0.774–0.777)	0.730 (0.729–0.732)
Retrospective (R2)	592,504	37,930,348	0.872 (0.872–0.873)	0.754 (0.754–0.755)	0.808 (0.807–0.809)	0.749 (0.748–0.749)
Retrospective (R3)	930,008	75,681,392	0.807 (0.806–0.807)	0.812 (0.811–0.812)	0.744 (0.743–0.744)	0.674 (0.674–0.675)
Prospective (all)	205,946	21,516,209	0.828 (0.827–0.829)	0.768 (0.767–0.769)	0.767 (0.766–0.768)	0.709 (0.708–0.710)
Prospective (P1)	8,036	1,270,931	0.855 (0.851–0.858)	0.734 (0.729–0.738)	0.768 (0.764–0.773)	0.735 (0.731–0.739)
Prospective (P2)	33,092	2,928,320	0.873 (0.872–0.875)	0.819 (0.817–0.821)	0.809 (0.807–0.811)	0.757 (0.755–0.760)
Prospective (P3)	164,818	17,316,958	0.817 (0.816–0.818)	0.774 (0.773–0.775)	0.758 (0.757–0.759)	0.697 (0.696–0.698)

Performance across subgroups in the retrospective validation (**Table 3**) demonstrated that eCARTv5 (AUROCs, 0.810–0.909) consistently outperformed eCARTv2 (AUROCs 0.708–0.804) and NEWS (AUROCs, 0.745–0.793), which outperformed MEWS (AUROCs, 0.672–0.726). Among the different age groups, the AUROC for eCARTv5 was highest in 18 to 33-year-old patients (0.861 [95% CI, 0.859–0.862]) and lowest in 65- to 78-year-old patients (0.822 [95% CI, 0.822–0.823]). In the subgroup analysis by race, the eCARTv5 AUROC was highest in the Native Hawaiian/Other Pacific Islander cohort (0.862 [95% CI, 0.854–0.870]) and lowest in the American Indian or Alaska Native cohort (0.814 [95% CI, 0.808–0.820]). eCARTv5 performance was slightly higher for female compared with male patients (0.844 [95% CI, 0.843–0.844] vs. 0.824 [95% CI, 0.824–0.825]) and was exceptionally high in obstetric encounters (0.909 [95% CI, 0.905–0.912]). Among the clinical conditions, performance was highest in patients with COVID-19 across all scores except eCARTv2 (which was highest in sepsis) and lowest in heart failure. Across all subgroups studied, eCARTv5 retained high discrimination (AUROC ≥0.81).

**TABLE 3.** Subgroup Analysis Results Showing the Area Under the Receiver Operating Characteristic Curve (AUROC) Values for Predicting Clinical Deterioration in the Full Retrospective Cohort By Risk Score and Subgroup

Category	Subgroup	Encounters, *n*	Observations, *n*	eCARTv5 AUROC (95% CI)	eCARTv2 AUROC (95% CI)	National Early Warning Score AUROC (95% CI)	Modified Early Warning Score AUROC (95% CI)
All	–	1,769,461	132,873,833	0.834 (0.834–0.835)	0.775 (0.775–0.776)	0.766 (0.766–0.767)	0.704 (0.703–0.704)
Age	18–33	232,353	10,934,645	0.861 (0.859–0.862)	0.769 (0.767–0.771)	0.770 (0.767–0.772)	0.726 (0.723–0.728)
	34–48	271,904	16,360,824	0.845 (0.844–0.846)	0.776 (0.774–0.777)	0.758 (0.757–0.760)	0.709 (0.707–0.710)
	49–64	475,033	37,818,597	0.827 (0.827–0.828)	0.764 (0.764–0.765)	0.755 (0.755–0.756)	0.702 (0.701–0.703)
	65–78	458,470	39,748,074	0.822 (0.822–0.823)	0.761 (0.760–0.761)	0.758 (0.757–0.759)	0.700 (0.700–0.701)
	≥79	331,701	28,011,693	0.832 (0.831–0.832)	0.775 (0.774–0.776)	0.776 (0.775–0.777)	0.716 (0.715–0.716)
Sex	Male	776,164	64,367,912	0.824 (0.824–0.825)	0.764 (0.764–0.765)	0.761 (0.760–0.761)	0.699 (0.699–0.700)
	Female	993,297	68,505,921	0.844 (0.843–0.844)	0.786 (0.785–0.787)	0.775 (0.774–0.775)	0.710 (0.709–0.711)
Race	American Indian or Alaska Native	6,468	486,615	0.814 (0.808–0.820)	0.741 (0.734–0.748)	0.746 (0.739–0.753)	0.672 (0.664–0.679)
	Asian/Mideast Indian	26,681	1,694,681	0.847 (0.844–0.850)	0.795 (0.791–0.798)	0.779 (0.775–0.782)	0.722 (0.718–0.725)
	Black/African American	252,982	20,057,696	0.831 (0.830–0.832)	0.776 (0.775–0.777)	0.769 (0.768–0.770)	0.707 (0.706–0.708)
	Native Hawaiian/Other Pacific Islander	2,496	177,513	0.862 (0.854–0.870)	0.794 (0.784–0.804)	0.772 (0.762–0.783)	0.709 (0.697–0.720)
	White	1,384,075	104,522,118	0.833 (0.833–0.834)	0.773 (0.772–0.773)	0.764 (0.764–0.765)	0.702 (0.702–0.703)
	Other/Unknown	96,759	5,935,210	0.858 (0.857–0.860)	0.804 (0.802–0.806)	0.785 (0.783–0.786)	0.724 (0.722–0.726)
Procedure	Surgical	539,875	53,320,071	0.816 (0.816–0.817)	0.763 (0.763–0.764)	0.745 (0.745–0.746)	0.690 (0.690–0.691)
	Obstetric	154,759	4,722,067	0.909 (0.905–0.912)	0.708 (0.701–0.716)	0.758 (0.751–0.766)	0.691 (0.684–0.697)
Clinical condition	Sepsis	639,802	72,055,421	0.836 (0.836–0.836)	0.779 (0.778–0.779)	0.774 (0.774–0.775)	0.714 (0.713–0.714)
	COVID-19	49,834	5,961,084	0.858 (0.857–0.859)	0.757 (0.756–0.759)	0.793 (0.792–0.794)	0.710 (0.709–0.712)
	Congestive heart failure	306,140	31,445,409	0.810 (0.809–0.810)	0.741 (0.741–0.742)	0.750 (0.749–0.750)	0.694 (0.694–0.695)
	Chronic obstructive pulmonary disease	443,263	38,638,180	0.824 (0.823–0.824)	0.760 (0.759–0.761)	0.757 (0.756–0.757)	0.698 (0.697–0.698)
Admission year	2009–2019	1,069,832	79,602,400	0.824 (0.823–0.824)	0.767 (0.767–0.768)	0.751 (0.751–0.752)	0.695 (0.694–0.695)
	2020–2023	699,629	53,271,433	0.849 (0.849–0.850)	0.786 (0.786–0.787)	0.788 (0.787–0.788)	0.716 (0.716–0.717)

**Figure 3.**
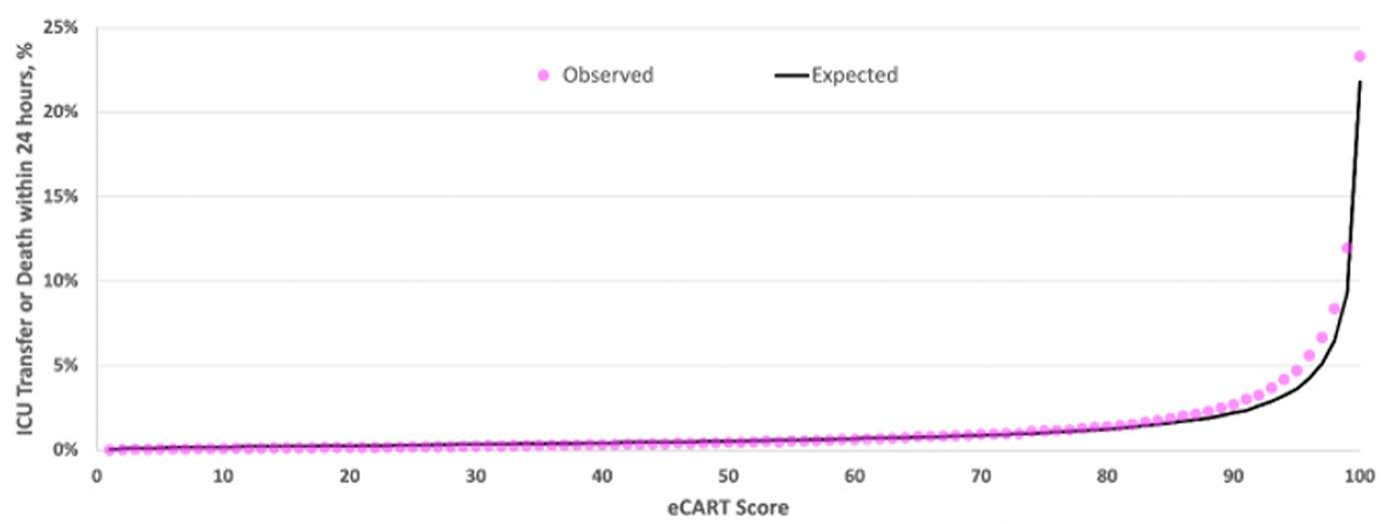
Calibration plot in the prospective validation cohort illustrating the observed and expected outcome rates across values of the eCARTv5 score.

In the prospective analysis, there were 21,516,209 scores calculated, and 7,303 encounters had clinical deterioration within 24 hours following an observation. Performance in the full prospective cohort was similar to the full retrospective cohort, with eCARTv5 (AUROC, 0.828 [95% CI, 0.827–0.829]) outperforming eCARTv2 (AUROC, 0.768 [95% CI, 0.767–0.769]), NEWS (AUROC, 0.767 [95% CI, 0.766–0.768]), and MEWS (AUROC, 0.709 [95% CI, 0.708–0.710]). Model calibration is shown in **Figure 3** and **Figure E2** (http://links.lww.com/CCX/B484), demonstrating close agreement between the observed and expected deterioration rates for lower values and then underprediction of risk at the higher scores (calibration intercept = 0.36 and calibration slope = 1.07). Event rates for NEWS and MEWS for each score value are shown in **Figures E3 and E4** (http://links.lww.com/CCX/B484). Finally, in a head-to-head comparison of the real-time device output scoring and the retrospectively calculated scores in a convenience sample of 162,335 prospective cohort encounters from P3, the device calculated 17,126,259 eCARTv5 scores in real-time in those encounters with a mean value of 53 (sd 28), while the retrospective extract generated 15,251,520 eCARTv5 scores with a mean of 49 (sd 29). The resulting AUROCs were slightly higher using the retrospective calculation methodology compared with the prospectively collected score [0.825 (0.824–0.826) vs. 0.817 (0.816–0.818)].

## DISCUSSION

In a large retrospective validation of nearly two million inpatient encounters with over 130 million calculated scores from three geographically distinct health systems in the United States, eCARTv5 outperformed MEWS, NEWS, and eCARTv2 for predicting clinical deterioration. Results were robust across age, sex, and race, with eCARTv5 performing better than the other scores in all subgroups. Further, eCARTv5 had consistently high discrimination in patients with heart failure, COPD, sepsis, and COVID-19, as well as in surgical and obstetric patients. This illustrates an important strength of developing an all-cause deterioration model because it enhances early identification across a wide range of patients instead of more narrowly developed models for specific conditions (e.g., sepsis). Performance was confirmed in a prospective study of over 200,000 admissions. These results, which constitute the largest validation of an early warning score to date, provide confidence that eCARTv5’s performance is strong and generalizable. Prospective implementation of eCARTv5 would lead to increased detection and decreased false alarm rates, which could improve patient outcomes and decrease alarm fatigue.

eCARTv2, which our group developed in 2014, included 269,999 admissions from three health systems in Illinois and used logistic regression (1). The AAM score developed by Kipnis and colleagues used a similar approach to predict unplanned ICU transfer in the Kaiser Permanente Northern California health system (2). Prospective implementation studies of both scores found an association with decreased mortality (8, 9). However, our group and others have found that more advanced machine learning methods, such as GBM, can outperform logistic regression for predicting clinical deterioration (13, 14). Further, including trends has also been shown to improve model discrimination (22). Therefore, in this new version of eCART, we used GBM with trends to optimize performance. In this article, we found that eCARTv5 consistently outperformed the prior version created using logistic regression (eCARTv2), which may be due to the use of GBM, the inclusion of trends, and/or the use of more contemporaneous data. The high performance of eCARTv5 is consistent with prior early warning scores developed using machine learning, including the Hospital-wide Alerting Via Electronic Noticeboard, which is a GBM model developed and then validated in four hospitals in the United Kingdom that outperformed other scoring systems based on logistic regression (14). The increased discrimination of these more advanced models can increase detection rates while limiting false positive alerts that can cause alarm fatigue.

While previously published studies have demonstrated that more advanced models can outperform standard tools, such as MEWS and NEWS, across the entire medical-surgical cohort (2, 13, 14), little is known regarding comparative performance across important patient subgroups. Therefore, in this study, we performed extensive subgroup analysis that included age, sex, race, and medical conditions. We found that eCARTv5 had high discrimination across all subgroups and consistently outperformed eCARTv2, MEWS, and NEWS. The highest performance was in the post-partum cohort, followed by age 18 to 33, Native Hawaiian and Other Pacific Islanders, and patients with COVID-19. Across all 19 tested subgroups, eCARTv5 maintained an AUROC of ≥0.81. To the best of our knowledge, this is the largest and most comprehensive subgroup analysis performed on early warning scores to date and demonstrates that eCARTv5 can be used across a wide range of hospitalized patients.

Although numerous predictive models have been developed, few have been implemented prospectively. An important step toward implementation is building the informatics infrastructure for score calculation and assessing performance during silent implementation. During prospective validation, we found that eCARTv5 had similar performance to the retrospective evaluation. These results are encouraging, given that data quality and timing can differ between prospective and retrospective data. The head-to-head comparison of retrospectively calculated eCARTv5 scores in the prospective cohort to the scores filed back to the EHR in real time demonstrated a 12% increase in number of scores as well as a slightly higher average score, associated with a slightly lower AUROC. There are several potential causes for this discrepancy, including the correction of previously filed values and backdating of vital signs. Nevertheless, these analyses increase confidence that eCARTv5 will continue to perform well during implementation studies. Furthermore, we demonstrate the feasibility of testing these models in environments for clinical operations integrated with Health Level Seven.

Our study has several limitations. First, GBM models are complex and difficult to interpret. Therefore, explainable machine learning approaches are needed to provide insights to clinicians regarding the variables that are driving an individual patient’s risk of deterioration. In addition, there are myriad other machine learning approaches available to develop prediction models, and numerous possible comparator scores that have been published. We chose GBM due to its excellent discrimination and calibration from prior publications, and NEWS and MEWS due to their widespread use across the country and around the world. Furthermore, we were not able to determine whether ICU transfers were unanticipated vs. expected, which could have impacted performance metrics aimed at identifying true clinical deterioration. Finally, it is also important to note that high model discrimination may not translate to improved patient outcomes. For outcomes to be improved, the model needs to prompt lifesaving actions by the bedside clinicians, and the outcome studied must be preventable. Therefore, prospective implementation of eCARTv5 is required to study its impact on patient care.

## CONCLUSIONS

In conclusion, we developed and validated a new GBM model, eCARTv5, which accurately identifies early clinical deterioration. Our model was validated retrospectively in a geographically diverse set of health systems and performed better than the NEWS, MEWS, and eCARTv2 across multiple subgroups. Prospective validation of eCARTv5 found similar performance, and these results served as the foundation for an FDA 510(k) clearance.

## Supplementary Material
