# Supplementary material for: Multicenter Development and Prospective Validation of eCARTv5: A Gradient-Boosted Machine-Learning Early Warning Score
Source: Crit Care Explor. 2025 Mar 26;7(4):e1232. doi: 10.1097/CCE.0000000000001232 (PMC11949291; doi:10.1097/CCE.0000000000001232)

**Title:** Multicenter Development and Prospective Validation of eCARTv5: A Gradient Boosted Machine Learning Early Warning Score

**Authors:** Matthew M. Churpek, MD, MPH, PhD, ATSF[1,2]; Kyle A. Carey, MPH[3]; Ashley Snyder, MPH[4]; Christopher J Winslow, MD[5]; Emily Gilbert, MD[6]; Nirav S Shah, MD, MPH[5]; Brian W. Patterson, MD, MPH[2,7]; Majid Afshar, MD, MSCR[1,2]; Alan Weiss, MD, MBA[7]; Devendra N. Amin, MD[7]; Deborah J. Rhodes, MD[8]; Dana P. Edelson, MD, MS[3,4]

**Affiliations:**
[1]Department of Medicine, University of Wisconsin-Madison, Madison, WI
[2]Department of Biostatistics and Medical Informatics, University of Wisconsin-Madison, Madison, WI
[3]Department of Medicine, University of Chicago, Chicago, IL
[4]AgileMD, San Francisco, CA
[5]Department of Medicine, Endeavor Health, Evanston, IL
[6]Department of Medicine, Loyola University Medical Center, Chicago, IL
[7]Department of Emergency Medicine, University of Wisconsin-Madison, Madison WI
[8]BayCare, Clearwater, FL
[9]Department of Medicine, Yale University, New Haven, CT

**Corresponding author:**
Matthew M Churpek, MD, MPH, PhD
Email: mchurpek@medicine.wisc.edu

**ONLINE DATA SUPPLEMENT**

## Table of Contents:


| Supplement Section | Page |
|---|---|
| **Supplemental Methods** | **3** |
| Additional Institutional Review Board Information | 3 |
| Model Development | 4 |
| Subgroup Definitions | 5 |
| **Table E1.** Date ranges by cohort. | **6** |
| **Table E2.** Variables included in the eCARTv5 model. | **7** |
| **Table E3.** Non-physiologic filters for predictor variables. | **8** |
| **Table E4.** Demographics by geographic location. | **9** |
| **Table E5.** Heatmap distribution of variable missingness at the level of each eCARTv5 score for the derivation health system cohort. | **10** |
| **Table E6.** Heatmap distribution of variable missingness at the level of each eCARTv5 score for the retrospective and prospective validation cohorts by health system cohorts. | **14** |
| **Table E7.** Model discrimination by cohort and hospital. | **18** |
| **Table E8.** Bootstrap model discrimination. | **21** |
| **Table E9.** Full retrospective cohort eCARTv5 test characteristics for the primary outcome of deterioration. | **22** |
| **Table E10.** Full retrospective cohort eCARTv2 test characteristics for the primary outcome of deterioration. | **27** |
| **Table E11.** Full retrospective cohort NEWS test characteristics for the primary outcome of deterioration. | **29** |
| **Table E12.** Full retrospective cohort MEWS test characteristics for the primary outcome of deterioration. | **30** |
| **Figure E1.** eCARTv5 partial plots. Partial dependence plots of the association between maximum respiratory rate in the prior 24 hours (A), delivered FiO2 (B), minimum systolic blood pressure in the prior 24 hours, and heart rate (D) and the risk of the outcome. | **31** |
| **Figure E2.** eCARTv5 calibration curve. | **32** |
| **Figure E3.** Outcome rate for different NEWS values. | **33** |
| **Figure E4.** Outcome rate for different MEWS values. | **34** |

## Supplemental Methods

### Additional Institutional Review Board Information

The study was approved by the following Institutional Review Boards (IRB) with a waiver of informed consent:

1. The University of Chicago Biological Sciences Division IRB (#18-0447; Title: Epidemiology and prediction of critical care interventions during rapid response team calls; Initial approval 6/20/2018; relevant amendment approved 10/8/2021.)
2. Loyola University Chicago Health Sciences Division IRB (#215437; Title: “Developing a clinical decision support tool for the identification, diagnosis, and treatment of critical illness in hospitalized patients”; Initial approval 12/17/2021; relevant amendments approved 03/07/2022 and 8/31/2022.)
3. NorthShore University HealthSystem Research Institute IRB (#EH16-210T; Title: “Sepsis Early Prediction and Subphenotype Illumination Study”; Initial approval 08/02/2016; relevant amendment approved 04/19/2021.)
4. The University of Wisconsin-Madison Minimal Risk Research IRB (#2019-1258; Title: “Predicting in-hospital clinical deterioration”; Initial approval 11/15/2019; relevant amendment approved 2/22/2021.)
5. BayCare Health System IRB
    a. (Retrospective Study: #2022.014-B.MPH; Title: “Validation of the eCART Score for Predicting Clinical Deterioration in Hospitalized Patients”; Initial Approval 2/23/2022)
    b. (Prospective Study: #2022.015-B.MPH; Title: ”A Rapid Diagnostic of Risk in Hospitalized Patients with COVID-19, Sepsis, and Other High-Risk Conditions

to Improve Outcomes and Critical Resource Allocation Using Machine Learning"; Initial approval 2/23/2022)

6. Yale Human Research Protection Program IRBs (#2000035317; Title: "Validation of the electronic cardiac arrest risk triage (ECART) score for predicting clinical deterioration in hospitalized adults"; Initial approval 6/2/2023.)

**Model Development**

A gradient boosted machine (GBM) model was developed to predict clinical deterioration in all adult patients hospitalized on the wards in the training data. GBM models are ensembles of decision trees where each tree is iteratively built to improve upon the errors of the previous trees. In order to avoid overfitting and to optimize the final model, hyperparameters were tuned in the training cohort using five-fold cross-validation to maximize the area under the receiver operating characteristic curve (AUROC). Specifically, the number of trees (1000, 1500, 2000), interaction depth (20, 30, 40), shrinkage (0.001, 0.01, 0.1), and the minimum observations in a node (2, 4) were optimized, and the combination with the highest cross-validation AUROC was chosen as the final model.

**Subgroup Definitions**

Subgroup analyses were performed in the retrospective validation cohort across patient demographics (age, sex, race), admission year, and clinical conditions (surgical, obstetric, sepsis, COVID-19, congestive heart failure (CHF), and chronic obstructive pulmonary disease (COPD). Surgical patients were identified using hospital location

data denoting an operating room location. Sepsis was defined using the suspicion of infection criteria published by Seymour et al. Specifically, a patient was identified as infected ("sepsis") based on culture and antibiotic orders if either 1) a body fluid culture was ordered within 24 hours of an antibiotic if the antibiotic order was first or 2) the patient received an antibiotic in the next 72 hours if the body fluid culture order was first. A patient was included in the sepsis subgroup if they met the above suspicion of infection criteria, with the antibiotic or culture (whichever came first) occurring while they were on the ward (and prior to a potential deterioration event). Importantly, this is a retrospective definition and may not reflect the level of suspicion of the clinicians at the time of the orders. COVID-19 was defined as either a positive COVID-19 test (PCR or antigen) or a COVID-19 diagnosis code during the admission. Obstetric patients were identified using diagnosis codes for birth or C-section in the current encounter. Patients presenting to the labor and delivery location but who were not later admitted to an inpatient ward were excluded from the analysis. COPD and CHF were defined using the Elixhauser criteria for these conditions based on diagnosis codes from the hospital encounter.

**Table E1.** Date ranges by cohort.*

| Cohort | Date Range |
|---|---|
| Derivation (D1) | 2008 - 2022 |
| Derivation (D2) | 2008 - 2018 |
| Derivation (D3) | 2006 - 2017 |
| Retrospective (R1) | 2009 - 2020 |
| Retrospective (R2) | 2017- 2023 |
| Retrospective (R3) | 2016 - 2022 |
| Prospective (P1) | 10/17/2023 - 03/20/2024 |
| Prospective (P2) | 10/02/2023 - 01/15/2024 |
| Prospective (P3) | 02/08/2023 - 12/31/2023 |

*Note that the derivation and retrospective cohorts were date-shifted for de-identification purposes, so exact dates are unavailable.

**Table E2.** Variables included in the eCARTv5 model.

| | | |
|---|---|---|
| Demographics | Age | Continuous |
| Vital signs | Temperature (C°), Heart Rate, Respiratory Rate, Systolic Blood Pressure (SBP), Diastolic Blood Pressure (DBP), O2 Saturation, Fraction of Inspired Oxygen (FiO2), AVPU, Disorientation (yes/no) | Continuous |
| Vital sign trends | Highest value in the last 24 hours of Heart Rate, Respiratory Rate, SBP, DBP, FiO2, AVPU (U=highest), and Disorientation (yes=highest) | Continuous |
| | Lowest value in last 24 hours of Heart Rate, SBP, DBP, O2 Saturation, and FiO2 | |
| | Mean over the last 24 hours of Heart rate, Respiratory Rate, SBP, O2 Saturation, and FiO2 | |
| | Standard Deviation over the last 24 hours of Temperature, Heart Rate, Respiratory Rate, SBP, DBP, and FiO2 | |
| | Slope over the last 24 hours of Heart Rate, Respiratory Rate, Temperature, SBP, DBP, and FiO2 | |
| Laboratory values | Basic Metabolic Panel [BMP]: Sodium, Chloride, Potassium, Bicarbonate (CO2), Anion Gap, Glucose, Calcium, Blood Urea Nitrogen (BUN), Serum Creatinine (SCr), Phosphate | Continuous |
| | Liver Function Test [LFT]: Total Protein, Albumin, Total Bilirubin, Aspartate Aminotransferase (AST/SGOT), Alkaline Phosphatase | |
| | Complete Blood Count [CBC]: White Blood Cells (WBC), Hemoglobin, Platelet Count, Bands, Eosinophils, Lymphocytes, Monocytes, Neutrophils | |
| | Blood Gas test: Arterial pH, Venous pH, Arterial Partial Pressure of Oxygen, Arterial Partial Pressure of Carbon Dioxide, Venous Partial Pressure of Carbon Dioxide | |
| | Other labs: Lactate, Magnesium, Lipase, International Normalized Ratio (INR), Mean Corpuscular Volume (MCV), Partial Thromboplastin Time (PTT), and Red Cell Distribution Width (RDW) | |
| Laboratory value trends | Change from last collected value of Potassium, SCr, BUN, CO2, Anion Gap, Glucose, Phosphate, WBC, Hemoglobin, and Platelet Count | Continuous |
| Nurse Documentation | Braden Scale (Activity, Friction and Shear, Mobility, Moisture, Nutrition, Sensory Perception, Total Score), Body Mass Index, Change from last recorded BMI | Continuous |
| Location | Prior Intensive Care Unit stay | Binary |
| Length of stay | Hours since admission until the current time point | Continuous |
| Time of Day | Hours since midnight of the current day | Continuous |
| Urinary Output | Sum of Urine Output over the last 24 hours | Continuous |

Abbreviations: AVPU = Alert, responds to Voice, responds to Pain, Unresponsive

**Table E3.** Non-physiologic filters for predictor variables.

| **Variable** | **Filter threshold to change to missing** |
|---|---|
| Temperature, °C | < 32 or > 44 |
| Respiratory rate, breaths per minute | < 1 or > 70 |
| Heart rate, beats per minute | < 1 or > 300 |
| Systolic blood pressure, mm Hg | < 30 or > 300 |
| Diastolic blood pressure, mm Hg | < 1 or > 250 |
| Peripheral oxygen saturation, % | < 11 or > 100 |
| $FiO_2$ delivered, % | <21 or >100 |
| White blood cells, x$10^9$/L | > 1000 |
| White blood cell components, % | <0 or >100 |
| Body mass Index, kg/$m^2$ | < 10 or > 200 |

**Table E4.** Demographics by geographic location.

| Patient Characteristics | Midwestern Cohort | Eastern Cohort | Southern Cohort |
|---|---|---|---|
| Hospitals, N | 8 | 7 | 13 |
| Encounters, N | 1,156,476 | 625,596 | 1,094,826 |
| Admission age, years, median (IQR) | 60 (45, 73) | 63 (43, 76) | 63 (47, 76) |
| Female sex | 635,471 (55%) | 359,389 (57%) | 624,057 (57%) |
| Race: American Indian or Alaska Native | 3,575 (0.3%) | 1,920 (0.3%) | 2,956 (0.3%) |
| Race: Asian/Mideast Indian | 27,319 (2%) | 12,345 (2%) | 13,279 (1%) |
| Race: Black/African American | 301,109 (26%) | 103,590 (17%) | 163,843 (15%) |
| Race: Pacific Islander/ Hawaiian Native | 997 (0.1%) | 1,270 (0.2%) | 1,119 (0.1%) |
| Race: White/Caucasian | 727,495 (63%) | 431,673 (69%) | 879,496 (80%) |
| Race: Other | 95,981 (8%) | 74,798 (12%) | 34,133 (3%) |
| Surgical | 413,800 (36%) | 218,709 (35%) | 268,784 (25%) |
| Obstetric | 66,124 (6%) | 85,544 (14%) | 81,338 (7%) |
| Sepsis | 323,964 (28%) | 226,288 (36%) | 407,497 (37%) |
| COVID-19 | 9,638 (1%) | 24,916 (4%) | 27,565 (3%) |
| Congestive heart failure | 164,503 (14%) | 135,616 (22%) | 181,888 (17%) |
| Chronic pulmonary disease | 199,108 (17%) | 173,911 (28%) | 274,107 (25%) |
| Length of stay, hours, median (IQR) | 72 (41, 131) | 90 (53, 159) | 61 (33, 107) |
| Ward to ICU transfer | 41,883 (4%) | 21,003 (3%) | 33,366 (3%) |
| Mortality | 14,526 (1%) | 13,894 (2%) | 10,840 (1%) |

**Table E5.** Heatmap distribution of variable missingness at the level of each eCARTv5 score for the derivation health system cohort.

| eCARTv5 variable | Derivation Cohort | | |
|---|---|---|---|
| | D1 | D2 | D3 |
| Temperature | 0.4% | 0.5% | 0.9% |
| Temperature, std dev | 2.7% | 3.4% | 6.0% |
| Temperature, slope | 2.7% | 3.4% | 6.0% |
| Heart Rate | 0.5% | 0.4% | 0.4% |
| Heart Rate, Maximum | 3.0% | 3.2% | 4.3% |
| Heart Rate, Mean | 3.0% | 3.2% | 4.3% |
| Heart Rate, Minimum | 3.0% | 3.2% | 4.3% |
| Heart Rate, std dev | 3.0% | 3.2% | 4.3% |
| Heart Rate, slope | 3.0% | 3.2% | 4.3% |
| Systolic Blood Pressure | 0.3% | 0.4% | 1.0% |
| Systolic Blood Pressure, Max | 2.8% | 3.2% | 5.6% |
| Systolic Blood Pressure, Mean | 2.8% | 3.2% | 5.6% |
| Systolic Blood Pressure, Min | 2.8% | 3.2% | 5.6% |
| Systolic Blood Pressure, Std dev | 2.8% | 3.2% | 5.6% |
| Systolic Blood Pressure, Slope | 2.8% | 3.2% | 5.6% |
| Diastolic Blood Pressure | 0.3% | 0.4% | 1.0% |
| Diastolic Blood Pressure, Max | 2.8% | 3.2% | 5.6% |
| Diastolic Blood Pressure, Min | 2.8% | 3.2% | 5.6% |
| Diastolic Blood Pressure, Std dev | 2.8% | 3.2% | 5.6% |
| Diastolic Blood Pressure, Slope | 2.8% | 3.2% | 5.6% |
| Respirations | 0.4% | 0.4% | 0.8% |
| Respirations, Max | 2.5% | 3.2% | 5.3% |
| Respirations, Mean | 2.5% | 3.2% | 5.3% |
| Respirations, Std dev | 2.5% | 3.2% | 5.3% |
| Respirations, Slope | 2.5% | 3.2% | 5.3% |

| eCARTv5 variable | Derivation Cohort | | |
|---|---|---|---|
| | D1 | D2 | D3 |
| Oxygen Saturation | 0.5% | 0.8% | 2.1% |
| Oxygen Saturation, Min | 2.8% | 6.4% | 14.9% |
| Oxygen Saturation, Mean | 2.8% | 6.4% | 14.9% |
| FiO2 | 3.1% | 6.0% | 29.2% |
| FiO2, Max | 10.6% | 18.4% | 47.0% |
| FiO2, Mean | 10.6% | 18.4% | 47.0% |
| FiO2, Min | 10.6% | 18.4% | 47.0% |
| FiO2, Std div | 10.6% | 18.4% | 47.0% |
| FiO2, Slope | 10.6% | 18.4% | 47.0% |
| BMI | 10.1% | 61.3% | 45.1% |
| BMI, delta | 37.9% | 88.1% | 74.2% |
| Urine Output | 0.0% | 0.0% | 0.0% |
| AVPU | 6.1% | 13.4% | 1.9% |
| AVPU, Least responsiveness (24hrs) | 13.2% | 68.1% | 7.4% |
| AVPU, Disoriented | 7.0% | 11.3% | 2.7% |
| AVPU, Ever disoriented (24hrs) | 16.2% | 44.8% | 9.1% |
| Braden Scale | 3.5% | 12.9% | 6.0% |
| Braden Scale, Sensory | 3.4% | 5.0% | 5.9% |
| Braden Scale, Moisture | 3.5% | 5.0% | 5.9% |
| Braden Scale, Activity | 3.4% | 5.1% | 5.9% |
| Braden Scale, Mobility | 3.4% | 5.0% | 5.9% |
| Braden Scale, Nutrition | 3.4% | 5.0% | 5.9% |
| Braden Scale, Friction | 3.5% | 5.1% | 6.0% |
| White Blood Cells | 5.5% | 8.2% | 7.4% |
| White Blood Cells, Delta | 21.1% | 26.9% | 21.6% |
| White Blood Cells, Neutrophils | 39.6% | 28.1% | 74.7% |
| White Blood Cells, Bands | 65.9% | 26.3% | 26.7% |

| eCARTv5 variable | Derivation Cohort | | |
|---|---|---|---|
| | D1 | D2 | D3 |
| White Blood Cells, Lymphocytes | 30.2% | 32.4% | 26.9% |
| White Blood Cells, Monocytes | 30.3% | 25.2% | 27.1% |
| White Blood Cells, Eosinophils | 35.0% | 27.1% | 28.7% |
| Hemoglobin | 5.4% | 7.8% | 6.1% |
| Hemoglobin, Delta | 19.4% | 26.1% | 21.8% |
| Hemoglobin, MCV | 5.5% | 8.2% | 6.8% |
| Hemoglobin, RDW | 5.6% | 8.2% | 6.8% |
| Platelets | 5.5% | 8.2% | 7.8% |
| Platelets, Delta | 21.0% | 27.0% | 25.1% |
| aPTT | 51.8% | 69.6% | 51.7% |
| INR | 38.1% | 51.7% | 40.1% |
| Glucose | 8.3% | 17.4% | 9.7% |
| Glucose, Delta | 21.0% | 36.0% | 24.2% |
| Sodium | 8.3% | 17.5% | 9.7% |
| Potassium | 8.4% | 17.4% | 9.7% |
| Potassium, Delta | 21.2% | 35.9% | 24.2% |
| Chloride | 8.3% | 17.5% | 9.7% |
| Carbon Dioxide | 8.3% | 17.2% | 8.2% |
| Carbon Dioxide, Delta | 21.1% | 35.3% | 22.8% |
| Anion Gap | 8.3% | 17.5% | 9.7% |
| Anion Gap, Delta | 21.1% | 36.0% | 24.3% |
| BUN | 8.3% | 17.3% | 10.1% |
| BUN, Delta | 21.0% | 36.0% | 25.0% |
| Creatinine | 8.3% | 17.2% | 9.9% |
| Creatinine, Delta | 21.0% | 35.9% | 24.9% |
| Calcium | 8.2% | 17.4% | 10.3% |
| Magnesium | 18.1% | 63.9% | 32.1% |
| Phosphate | 19.6% | 74.9% | 37.9% |

| eCARTv5 variable | Derivation Cohort | | |
|---|---|---|---|
| | D1 | D2 | D3 |
| Phosphate, Delta | 34.3% | 84.2% | 50.6% |
| Protein | 34.8% | 54.8% | 41.9% |
| Albumin | 34.2% | 53.3% | 41.0% |
| Bilirubin, Total | 34.8% | 55.0% | 41.9% |
| Alk Phos | 34.9% | 55.0% | 42.0% |
| AST / SGOT | 35.0% | 54.8% | 41.9% |
| Lactate | 94.5% | 97.3% | 95.9% |
| Lipase | 81.9% | 83.9% | 85.7% |
| ABG pH | 98.8% | 98.7% | 87.3% |
| ABG pCO2 | 98.7% | 98.7% | 98.3% |
| ABG pO2 | 98.7% | 98.7% | 98.3% |
| VBG pH | 98.2% | 99.8% | 99.7% |
| VBG pCO2 | 98.2% | 99.8% | 99.7% |
| Age on admission | 0.0% | 0.0% | 0.0% |
| Length of stay | 0.0% | 0.0% | 0.0% |
| ICU this admission | 0.0% | 0.0% | 0.0% |
| Time of day | 0.0% | 0.0% | 0.0% |

**Table E6.** Heatmap distribution of variable missingness at the level of each eCARTv5 score for the retrospective and prospective validation cohorts by health system cohorts.

| eCARTv5 variable | Health System 1 | | Health System 2 | | Health System 3 | | |
|---|---|---|---|---|---|---|---|
| | Retrospective, R1 | Prospective, P1 | Retrospective, R2 | Prospective, P2 | Retrospective, R3 | Prospective (Real Time Score), P3 | Retrospectively Calculated Prospective, P3(r) |
| Temperature | 0.2% | 0.4% | 0.1% | 0.1% | 0.1% | 0.2% | 0.1% |
| Temperature, std dev | 4.3% | 3.9% | 1.6% | 2.6% | 1.9% | 3.6% | 1.9% |
| Temperature, slope | 4.3% | 3.9% | 1.6% | 2.6% | 1.9% | 3.6% | 1.9% |
| Heart Rate | 0.1% | 0.0% | 0.1% | 0.0% | 0.1% | 0.0% | 0.1% |
| Heart Rate, Maximum | 2.6% | 1.4% | 1.0% | 1.4% | 1.0% | 0.7% | 0.7% |
| Heart Rate, Mean | 2.6% | 1.4% | 1.0% | 1.4% | 1.0% | 0.7% | 0.7% |
| Heart Rate, Minimum | 2.6% | 1.4% | 1.0% | 1.4% | 1.0% | 0.7% | 0.7% |
| Heart Rate, std dev | 2.6% | 1.4% | 1.0% | 1.4% | 1.0% | 0.7% | 0.7% |
| Heart Rate, slope | 2.6% | 1.4% | 1.0% | 1.4% | 1.0% | 0.7% | 0.7% |
| Systolic Blood Pressure | 0.2% | 0.0% | 0.2% | 0.1% | 0.0% | 0.0% | 0.0% |
| Systolic Blood Pressure, Max | 3.3% | 1.6% | 1.6% | 2.0% | 0.6% | 0.6% | 0.5% |
| Systolic Blood Pressure, Mean | 3.3% | 1.6% | 1.6% | 2.0% | 0.6% | 0.6% | 0.5% |
| Systolic Blood Pressure, Min | 3.3% | 1.6% | 1.6% | 2.0% | 0.6% | 0.6% | 0.5% |
| Systolic Blood Pressure, Std dev | 3.3% | 1.6% | 1.6% | 2.0% | 0.6% | 0.6% | 0.5% |
| Systolic Blood Pressure, Slope | 3.3% | 1.6% | 1.6% | 2.0% | 0.6% | 0.6% | 0.5% |
| Diastolic Blood Pressure | 0.2% | 0.0% | 0.2% | 0.1% | 0.0% | 0.0% | 0.0% |
| Diastolic Blood Pressure, Max | 3.3% | 1.6% | 1.6% | 2.0% | 0.6% | 0.6% | 0.5% |
| Diastolic Blood Pressure, Min | 3.3% | 1.6% | 1.6% | 2.0% | 0.6% | 0.6% | 0.5% |
| Diastolic Blood Pressure, Std dev | 3.3% | 1.6% | 1.6% | 2.0% | 0.6% | 0.6% | 0.5% |
| Diastolic Blood Pressure, Slope | 3.3% | 1.6% | 1.6% | 2.0% | 0.6% | 0.6% | 0.5% |
| Respirations | 0.3% | 0.4% | 0.1% | 0.1% | 0.2% | 0.3% | 0.3% |
| Respirations, Max | 2.8% | 2.5% | 1.4% | 2.2% | 7.4% | 11.3% | 11.0% |
| Respirations, Mean | 2.8% | 2.5% | 1.4% | 2.2% | 7.4% | 11.3% | 11.0% |
| Respirations, Std dev | 2.8% | 2.5% | 1.4% | 2.2% | 7.4% | 11.3% | 11.0% |
| Respirations, Slope | 2.8% | 2.5% | 1.4% | 2.2% | 7.4% | 11.3% | 11.0% |
| Oxygen Saturation | 0.4% | 0.1% | 0.2% | 0.1% | 0.2% | 0.1% | 0.1% |
| Oxygen Saturation, Min | 4.4% | 1.6% | 1.3% | 1.6% | 1.6% | 0.9% | 0.9% |
| Oxygen Saturation, Mean | 4.4% | 1.6% | 1.3% | 1.6% | 1.6% | 0.9% | 0.9% |

| eCARTv5 variable | Health System 1 | | Health System 2 | | Health System 3 | | |
|---|---|---|---|---|---|---|---|
| | Retrospective, R1 | Prospective, P1 | Retrospective, R2 | Prospective, P2 | Retrospective, R3 | Prospective (Real Time Score), P3 | Retrospectively Calculated Prospective, P3(r) |
| FiO2 | 0.9% | 1.0% | 1.3% | 1.2% | 0.2% | 0.8% | 0.2% |
| FiO2, Max | 8.6% | 8.6% | 7.7% | 8.6% | 4.5% | 5.4% | 2.4% |
| FiO2, Mean | 8.6% | 8.6% | 7.7% | 8.6% | 4.5% | 5.4% | 2.4% |
| FiO2, Min | 8.6% | 8.6% | 7.7% | 8.6% | 4.5% | 5.4% | 2.4% |
| FiO2, Std div | 8.6% | 8.6% | 7.7% | 8.6% | 4.5% | 5.4% | 2.4% |
| FiO2, Slope | 8.6% | 8.6% | 7.7% | 8.7% | 4.5% | 5.4% | 2.4% |
| BMI | 4.3% | 4.4% | 10.8% | 15.8% | 11.6% | 8.5% | 8.3% |
| BMI, delta | 38.1% | 21.0% | 44.8% | 56.4% | 46.5% | 11.3% | 39.7% |
| Urine Output | 0.0% | 0.0% | 0.0% | 0.0% | 0.0% | 0.0% | 0.0% |
| AVPU | 1.4% | 12.7% | 2.7% | 6.2% | 0.3% | 0.3% | 0.1% |
| AVPU, Least responsiveness (24hrs) | 8.2% | 44.3% | 19.6% | 33.2% | 4.8% | 11.8% | 3.3% |
| AVPU, Disoriented | 8.9% | 3.2% | 1.4% | 40.1% | 0.7% | 9.7% | 0.4% |
| AVPU, Ever disoriented (24hrs) | 20.5% | 9.1% | 16.3% | 67.9% | 8.8% | 49.6% | 7.7% |
| Braden Scale | 2.1% | 5.2% | 1.6% | 6.3% | 6.2% | 6.4% | 3.5% |
| Braden Scale, Sensory | 2.1% | 5.2% | 1.5% | 6.2% | 5.9% | 6.4% | 3.5% |
| Braden Scale, Moisture | 1.9% | 5.2% | 1.5% | 6.3% | 5.9% | 6.4% | 3.5% |
| Braden Scale, Activity | 1.6% | 5.2% | 1.5% | 6.3% | 5.9% | 6.4% | 3.5% |
| Braden Scale, Mobility | 2.1% | 5.2% | 1.5% | 6.3% | 5.9% | 6.4% | 3.5% |
| Braden Scale, Nutrition | 2.1% | 5.2% | 1.5% | 6.3% | 5.9% | 6.4% | 3.5% |
| Braden Scale, Friction | 2.1% | 5.2% | 1.5% | 6.7% | 5.9% | 6.4% | 3.5% |
| White Blood Cells | 10.9% | 5.7% | 11.1% | 3.5% | 4.1% | 8.8% | 2.0% |
| White Blood Cells, Delta | 31.2% | 23.8% | 22.6% | 19.1% | 28.9% | 33.8% | 24.7% |
| White Blood Cells, Neutrophils | 25.3% | 25.2% | 2.9% | 6.5% | 6.1% | 13.1% | 7.0% |
| White Blood Cells, Bands | 92.6% | 28.3% | 81.8% | 7.4% | 86.7% | 93.5% | 93.1% |
| White Blood Cells, Lymphocytes | 25.3% | 25.2% | 4.2% | 6.5% | 6.1% | 13.2% | 7.1% |
| White Blood Cells, Monocytes | 25.3% | 25.2% | 4.2% | 6.5% | 6.2% | 13.3% | 7.2% |
| White Blood Cells, Eosinophils | 25.3% | 25.2% | 4.3% | 6.6% | 10.1% | 19.2% | 12.3% |
| Hemoglobin | 10.4% | 4.8% | 2.3% | 3.4% | 3.5% | 8.5% | 1.7% |
| Hemoglobin, Delta | 30.3% | 20.1% | 15.8% | 18.8% | 27.3% | 32.5% | 22.5% |
| Hemoglobin, MCV | 12.0% | 5.7% | 2.5% | 3.5% | 4.1% | 8.9% | 2.0% |
| Hemoglobin, RDW | 12.0% | 5.8% | 2.9% | 3.6% | 4.1% | 8.9% | 2.0% |
| Platelets | 10.7% | 5.6% | 2.5% | 3.5% | 4.1% | 8.6% | 2.0% |

| eCARTv5 variable | Health System 1 | | Health System 2 | | Health System 3 | | |
|---|---|---|---|---|---|---|---|
| | Retrospective, R1 | Prospective, P1 | Retrospective, R2 | Prospective, P2 | Retrospective, R3 | Prospective (Real Time Score), P3 | Retrospectively Calculated Prospective, P3(r) |
| Platelets, Delta | 31.3% | 23.0% | 16.1% | 19.1% | 28.9% | 33.7% | 24.7% |
| aPTT | 71.3% | 70.2% | 59.0% | 63.1% | 68.8% | 76.1% | 75.2% |
| INR | 39.3% | 40.2% | 45.4% | 49.3% | 48.5% | 54.8% | 52.7% |
| Glucose | 10.5% | 5.9% | 6.5% | 6.2% | 11.1% | 7.0% | 6.0% |
| Glucose, Delta | 28.7% | 20.5% | 16.6% | 20.3% | 33.9% | 31.7% | 27.0% |
| Sodium | 9.3% | 5.4% | 6.5% | 6.1% | 5.2% | 2.9% | 2.7% |
| Potassium | 8.6% | 5.1% | 6.6% | 6.3% | 5.2% | 3.0% | 2.7% |
| Potassium, Delta | 25.6% | 18.9% | 16.8% | 19.7% | 26.9% | 25.9% | 22.3% |
| Chloride | 9.5% | 5.5% | 6.7% | 6.1% | 5.2% | 4.4% | 2.7% |
| Carbon Dioxide | 9.4% | 5.4% | 6.6% | 6.0% | 11.0% | 6.8% | 5.7% |
| Carbon Dioxide, Delta | 26.8% | 18.5% | 15.7% | 17.8% | 33.5% | 30.5% | 25.7% |
| Anion Gap | 9.6% | 5.5% | 6.7% | 6.2% | 11.1% | 6.9% | 5.9% |
| Anion Gap, Delta | 28.0% | 21.6% | 17.5% | 19.7% | 33.8% | 32.0% | 26.5% |
| BUN | 10.2% | 6.4% | 6.4% | 6.0% | 5.2% | 4.4% | 2.7% |
| BUN, Delta | 28.8% | 22.1% | 16.7% | 19.5% | 27.0% | 28.2% | 22.5% |
| Creatinine | 9.1% | 4.9% | 6.5% | 6.1% | 11.1% | 7.0% | 6.0% |
| Creatinine, Delta | 27.2% | 19.6% | 17.7% | 20.2% | 33.7% | 31.5% | 26.9% |
| Calcium | 13.3% | 7.9% | 6.8% | 6.2% | 8.1% | 4.5% | 3.9% |
| Magnesium | 35.8% | 22.0% | 31.5% | 30.3% | 47.8% | 41.1% | 38.9% |
| Phosphate | 51.8% | 42.0% | 51.6% | 49.3% | 74.2% | 79.9% | 78.4% |
| Phosphate, Delta | 66.8% | 58.0% | 66.1% | 64.5% | 86.9% | 89.9% | 89.0% |
| Protein | 55.1% | 38.4% | 29.2% | 26.4% | 22.8% | 20.0% | 19.6% |
| Albumin | 48.7% | 35.0% | 27.3% | 24.6% | 22.6% | 19.7% | 19.2% |
| Bilirubin, Total | 45.7% | 34.3% | 25.8% | 24.5% | 22.8% | 20.0% | 19.5% |
| Alk Phos | 47.8% | 34.7% | 25.8% | 23.4% | 22.8% | 20.0% | 19.6% |
| AST / SGOT | 44.4% | 33.1% | 25.8% | 24.2% | 23.0% | 20.1% | 19.7% |
| Lactate | 95.0% | 90.9% | 92.6% | 90.9% | 94.0% | 89.7% | 92.1% |
| Lipase | 81.5% | 79.6% | 72.2% | 75.1% | 79.8% | 81.1% | 81.1% |
| ABG pH | 97.6% | 97.9% | 97.9% | 98.2% | 98.0% | 97.1% | 97.6% |
| ABG pCO2 | 97.6% | 97.9% | 97.6% | 97.3% | 98.0% | 97.2% | 97.5% |
| ABG pO2 | 97.6% | 97.9% | 97.9% | 98.2% | 98.0% | 97.2% | 97.6% |
| VBG pH | 98.3% | 94.8% | 97.0% | 98.9% | 99.8% | 99.2% | 99.4% |

| eCARTv5 variable | Health System 1 | | Health System 2 | | Health System 3 | | |
|---|---|---|---|---|---|---|---|
| | Retrospective, R1 | Prospective, P1 | Retrospective, R2 | Prospective, P2 | Retrospective, R3 | Prospective (Real Time Score), P3 | Retrospectively Calculated Prospective, P3(r) |
| VBG pCO2 | 98.3% | 94.8% | 97.0% | 98.9% | 99.8% | 99.7% | 99.4% |
| Age on admission | 0.0% | 0.0% | 0.0% | 0.0% | 0.0% | 0.0% | 0.0% |
| Length of stay | 0.0% | 0.0% | 0.0% | 0.0% | 0.0% | 0.0% | 0.0% |
| ICU this admission | 0.0% | 0.0% | 0.0% | 0.0% | 0.0% | 0.0% | 0.0% |
| Time of day | 0.0% | 0.0% | 0.0% | 0.0% | 0.0% | 0.0% | 0.0% |

**Table E7.** Model discrimination by cohort and hospital. Each model column presents the area under the receiver operating characteristic curve values and 95% confidence intervals for each score.

| Cohort | Hospital | Encounters, n | Observations, n | eCARTv5 | eCARTv2 | NEWS | MEWS |
|---|---|---|---|---|---|---|---|
| Retrospective (R1) | 1 | 246,949 | 19,262,093 | 0.861 (0.861, 0.862) | 0.789 (0.788, 0.790) | 0.775 (0.774, 0.777) | 0.730 (0.729, 0.732) |
| Retrospective (R2) | 1 | 106,572 | 6,215,346 | 0.863 (0.862, 0.865) | 0.821 (0.820, 0.823) | 0.804 (0.802, 0.806) | 0.752 (0.750, 0.753) |
| Retrospective (R2) | 2 | 57,818 | 2,282,704 | 0.886 (0.884, 0.889) | 0.822 (0.818, 0.825) | 0.822 (0.818, 0.825) | 0.755 (0.751, 0.759) |
| Retrospective (R2) | 3 | 63,087 | 4,213,365 | 0.871 (0.870, 0.873) | 0.815 (0.813, 0.816) | 0.799 (0.797, 0.801) | 0.754 (0.752, 0.756) |
| Retrospective (R2) | 4 | 10,586 | 584,901 | 0.843 (0.839, 0.847) | 0.791 (0.786, 0.796) | 0.780 (0.776, 0.785) | 0.714 (0.709, 0.719) |
| Retrospective (R2) | 5 | 97,525 | 6,526,370 | 0.875 (0.873, 0.876) | 0.814 (0.813, 0.816) | 0.821 (0.820, 0.823) | 0.755 (0.753, 0.757) |
| Retrospective (R2) | 6 | 16,187 | 779,228 | 0.842 (0.839, 0.846) | 0.766 (0.762, 0.770) | 0.762 (0.758, 0.766) | 0.719 (0.715, 0.723) |
| Retrospective (R2) | 7 | 240,729 | 17,328,434 | 0.874 (0.874, 0.875) | 0.812 (0.811, 0.813) | 0.809 (0.808, 0.810) | 0.745 (0.744, 0.746) |
| Retrospective (R3) | 1 | 19,019 | 1,422,002 | 0.777 (0.773, 0.781) | 0.739 (0.735, 0.743) | 0.717 (0.713, 0.721) | 0.666 (0.662, 0.670) |
| Retrospective (R3) | 2 | 101,598 | 7,304,165 | 0.812 (0.811, 0.813) | 0.769 (0.767, 0.770) | 0.762 (0.761, 0.764) | 0.701 (0.699, 0.703) |
| Retrospective (R3) | 3 | 17,268 | 1,220,928 | 0.836 (0.831, 0.840) | 0.817 (0.812, 0.821) | 0.801 (0.796, 0.805) | 0.723 (0.718, 0.729) |
| Retrospective (R3) | 4 | 133,395 | 8,958,784 | 0.827 (0.826, 0.829) | 0.765 (0.763, 0.767) | 0.758 (0.757, 0.760) | 0.690 (0.688, 0.691) |

| | | | | | | | |
|---|---|---|---|---|---|---|---|
| Retrospective (R3) | 5 | 60,085 | 4,666,948 | 0.831 (0.829, 0.833) | 0.781 (0.779, 0.783) | 0.766 (0.763, 0.768) | 0.681 (0.679, 0.684) |
| Retrospective (R3) | 6 | 122,869 | 11,377,852 | 0.789 (0.788, 0.790) | 0.749 (0.748, 0.751) | 0.733 (0.732, 0.735) | 0.673 (0.671, 0.674) |
| Retrospective (R3) | 7 | 40,157 | 2,979,367 | 0.778 (0.776, 0.781) | 0.734 (0.731, 0.736) | 0.711 (0.708, 0.714) | 0.646 (0.643, 0.649) |
| Retrospective (R3) | 8 | 142,506 | 16,165,025 | 0.805 (0.804, 0.806) | 0.743 (0.742, 0.744) | 0.741 (0.740, 0.742) | 0.675 (0.674, 0.676) |
| Retrospective (R3) | 9 | 54,755 | 4,128,150 | 0.810 (0.808, 0.811) | 0.747 (0.745, 0.749) | 0.739 (0.737, 0.741) | 0.674 (0.672, 0.677) |
| Retrospective (R3) | 10 | 72,569 | 5,261,906 | 0.768 (0.765, 0.770) | 0.722 (0.720, 0.725) | 0.706 (0.703, 0.709) | 0.648 (0.646, 0.651) |
| Retrospective (R3) | 11 | 33,094 | 1,347,721 | 0.847 (0.821, 0.873) | 0.657 (0.614, 0.700) | 0.621 (0.573, 0.668) | 0.625 (0.586, 0.664) |
| Retrospective (R3) | 12 | 124,139 | 10,650,197 | 0.795 (0.793, 0.796) | 0.722 (0.721, 0.723) | 0.730 (0.728, 0.731) | 0.653 (0.652, 0.655) |
| Retrospective (R3) | 13 | 8,554 | 198,347 | 0.793 (0.763, 0.823) | 0.743 (0.714, 0.773) | 0.767 (0.735, 0.799) | 0.596 (0.547, 0.644) |
| Prospective (P1) | 1 | 8,036 | 1,270,931 | 0.855 (0.851, 0.858) | 0.734 (0.729, 0.738) | 0.768 (0.764, 0.773) | 0.735 (0.731, 0.739) |
| Prospective (P2) | 1 | 5,317 | 513,960 | 0.860 (0.857, 0.864) | 0.816 (0.812, 0.821) | 0.807 (0.803, 0.812) | 0.766 (0.761, 0.771) |
| Prospective (P2) | 2 | 3,364 | 181,323 | 0.947 (0.940, 0.954) | 0.891 (0.879, 0.902) | 0.881 (0.867, 0.895) | 0.801 (0.784, 0.817) |
| Prospective (P2) | 3 | 4,273 | 320,389 | 0.861 (0.856, 0.866) | 0.837 (0.832, 0.841) | 0.812 (0.806, 0.817) | 0.767 (0.761, 0.773) |
| Prospective (P2) | 4 | 1,350 | 75,584 | 0.813 (0.801, 0.825) | 0.760 (0.745, 0.774) | 0.772 (0.759, 0.785) | 0.738 (0.725, 0.751) |
| Prospective (P2) | 5 | 6,551 | 589,740 | 0.892 (0.887, 0.897) | 0.828 (0.822, 0.833) | 0.829 (0.823, 0.836) | 0.765 (0.757, 0.772) |

| | | | | | | | |
|---|---|---|---|---|---|---|---|
| Prospective (P2) | 6 | 1,205 | 77,914 | 0.872 (0.862, 0.882) | 0.750 (0.735, 0.766) | 0.788 (0.774, 0.802) | 0.780 (0.767, 0.793) |
| Prospective (P2) | 7 | 11,032 | 1,169,410 | 0.874 (0.871, 0.876) | 0.821 (0.818, 0.824) | 0.798 (0.795, 0.802) | 0.738 (0.734, 0.742) |
| Prospective (P3) | 1 | 4,301 | 376,247 | 0.778 (0.772, 0.784) | 0.746 (0.739, 0.753) | 0.689 (0.682, 0.696) | 0.641 (0.634, 0.648) |
| Prospective (P3) | 2 | 21,834 | 2,163,745 | 0.823 (0.821, 0.825) | 0.772 (0.770, 0.775) | 0.769 (0.767, 0.772) | 0.704 (0.701, 0.707) |
| Prospective (P3) | 3 | 4,942 | 491,675 | 0.808 (0.803, 0.814) | 0.795 (0.790, 0.801) | 0.733 (0.727, 0.740) | 0.692 (0.686, 0.699) |
| Prospective (P3) | 4 | 26,499 | 3,038,030 | 0.822 (0.820, 0.824) | 0.770 (0.767, 0.772) | 0.769 (0.766, 0.771) | 0.712 (0.710, 0.715) |
| Prospective (P3) | 5 | 9,511 | 988,707 | 0.794 (0.790, 0.798) | 0.740 (0.735, 0.744) | 0.744 (0.740, 0.748) | 0.678 (0.674, 0.682) |
| Prospective (P3) | 6 | 22,899 | 2,767,216 | 0.826 (0.823, 0.828) | 0.781 (0.778, 0.783) | 0.758 (0.755, 0.760) | 0.702 (0.699, 0.705) |
| Prospective (P3) | 7 | 452 | 48,354 | 0.711 (0.698, 0.724) | 0.702 (0.690, 0.715) | 0.626 (0.613, 0.639) | 0.614 (0.601, 0.627) |
| Prospective (P3) | 8 | 20,752 | 2,570,565 | 0.854 (0.852, 0.857) | 0.799 (0.796, 0.803) | 0.803 (0.800, 0.807) | 0.736 (0.732, 0.739) |
| Prospective (P3) | 9 | 10,518 | 946,875 | 0.800 (0.796, 0.803) | 0.759 (0.755, 0.763) | 0.745 (0.741, 0.749) | 0.683 (0.679, 0.687) |
| Prospective (P3) | 10 | 16,629 | 1,397,404 | 0.811 (0.807, 0.816) | 0.781 (0.777, 0.786) | 0.749 (0.744, 0.754) | 0.699 (0.694, 0.704) |
| Prospective (P3) | 11 | 4,229 | 302,150 | 0.992 (0.987, 0.996) | 0.985 (0.980, 0.990) | 0.977 (0.966, 0.987) | 0.970 (0.956, 0.983) |
| Prospective (P3) | 12 | 20,776 | 2,158,184 | 0.773 (0.770, 0.776) | 0.743 (0.740, 0.746) | 0.724 (0.721, 0.727) | 0.671 (0.668, 0.674) |
| Prospective (P3) | 13 | 1,476 | 67,806 | 0.836 (0.805, 0.866) | 0.300 (0.221, 0.379) | 0.403 (0.348, 0.459) | 0.495 (0.456, 0.534) |

**Table E8.** Bootstrap model discrimination. Each column shows the score area under the receiver operating characteristic curve values and 95% confidence intervals.

| Cohort | Encounters, n | eCARTv5 AUROC (95% CI) | eCARTv2 AUROC (95% CI) | NEWS AUROC (95% CI) | MEWS AUROC (95% CI) |
|---|---|---|---|---|---|
| **Retrospective (All)** | 1,769,461 | 0.877 (0.875, 0.879) | 0.819 (0.816, 0.821) | 0.795 (0.792, 0.797) | 0.737 (0.735, 0.740) |
| **•Retrospective (R1)** | 246,949 | 0.882 (0.878, 0.887) | 0.803 (0.798, 0.809) | 0.790 (0.784, 0.797) | 0.745 (0.737, 0.751) |
| **•Retrospective (R2)** | 592,504 | 0.843 (0.841, 0.845) | 0.793 (0.791, 0.797) | 0.765 (0.762, 0.768) | 0.697 (0.693, 0.701) |
| **•Retrospective (R3)** | 930,008 | 0.917 (0.914, 0.919) | 0.860 (0.857, 0.863) | 0.840 (0.836, 0.843) | 0.787 (0.784, 0.792) |
| **Prospective (All)** | 205,946 | 0.858 (0.852, 0.863) | 0.791 (0.784, 0.796) | 0.782 (0.776, 0.787) | 0.729 (0.721, 0.736) |
| **•Prospective (P1)** | 8,036 | 0.872 (0.849, 0.892) | 0.752 (0.716, 0.778) | 0.791 (0.757, 0.817) | 0.756 (0.728, 0.787) |
| **•Prospective (P2)** | 33,092 | 0.843 (0.837, 0.850) | 0.797 (0.791, 0.803) | 0.768 (0.760, 0.774) | 0.710 (0.701, 0.718) |
| **•Prospective (P3)** | 164,818 | 0.915 (0.902, 0.924) | 0.853 (0.841, 0.864) | 0.850 (0.834, 0.864) | 0.807 (0.788, 0.824) |

Abbreviations: AUROC = area under the receiver operating characteristic curve

**Table E9**. Full retrospective cohort eCARTv5 test characteristics for the primary outcome of deterioration (N=1,769,461 encounters, n=132,873,833 observations).

| eCARTv5 | Sensitivity | Specificity | Positive Predictive Value | Negative Predictive Value |
|---|---|---|---|---|
| 0 | 100.0% (100.0%, 100.0%) | 0.0% (0.0%, 0.0%) | 1.3% (1.3%, 1.3%) | N/A |
| 1 | 100.0% (100.0%, 100.0%) | 1.6% (1.6%, 1.6%) | 1.3% (1.3%, 1.3%) | 100.0% (100.0%, 100.0%) |
| 2 | 99.9% (99.9%, 99.9%) | 2.6% (2.6%, 2.6%) | 1.3% (1.3%, 1.3%) | 100.0% (99.9%, 100.0%) |
| 3 | 99.8% (99.8%, 99.8%) | 3.9% (3.9%, 3.9%) | 1.4% (1.4%, 1.4%) | 99.9% (99.9%, 99.9%) |
| 4 | 99.7% (99.7%, 99.7%) | 4.9% (4.9%, 4.9%) | 1.4% (1.4%, 1.4%) | 99.9% (99.9%, 99.9%) |
| 5 | 99.6% (99.6%, 99.6%) | 6.0% (6.0%, 6.0%) | 1.4% (1.4%, 1.4%) | 99.9% (99.9%, 99.9%) |
| 6 | 99.5% (99.5%, 99.5%) | 7.1% (7.1%, 7.1%) | 1.4% (1.4%, 1.4%) | 99.9% (99.9%, 99.9%) |
| 7 | 99.4% (99.4%, 99.4%) | 8.3% (8.3%, 8.3%) | 1.4% (1.4%, 1.4%) | 99.9% (99.9%, 99.9%) |
| 8 | 99.3% (99.3%, 99.3%) | 8.9% (8.9%, 8.9%) | 1.4% (1.4%, 1.4%) | 99.9% (99.9%, 99.9%) |
| 9 | 99.2% (99.1%, 99.2%) | 10.1% (10.1%, 10.1%) | 1.4% (1.4%, 1.4%) | 99.9% (99.9%, 99.9%) |
| 10 | 99.0% (99.0%, 99.0%) | 11.4% (11.4%, 11.4%) | 1.5% (1.5%, 1.5%) | 99.9% (99.9%, 99.9%) |
| 11 | 98.8% (98.8%, 98.8%) | 12.6% (12.6%, 12.6%) | 1.5% (1.5%, 1.5%) | 99.9% (99.9%, 99.9%) |
| 12 | 98.7% (98.7%, 98.8%) | 13.2% (13.2%, 13.2%) | 1.5% (1.5%, 1.5%) | 99.9% (99.9%, 99.9%) |
| 13 | 98.6% (98.5%, 98.6%) | 14.4% (14.4%, 14.4%) | 1.5% (1.5%, 1.5%) | 99.9% (99.9%, 99.9%) |
| 14 | 98.4% (98.4%, 98.4%) | 15.6% (15.6%, 15.7%) | 1.5% (1.5%, 1.5%) | 99.9% (99.9%, 99.9%) |
| 15 | 98.3% (98.3%, 98.3%) | 16.2% (16.2%, 16.3%) | 1.5% (1.5%, 1.5%) | 99.9% (99.9%, 99.9%) |
| 16 | 98.1% (98.1%, 98.1%) | 17.4% (17.4%, 17.4%) | 1.6% (1.6%, 1.6%) | 99.9% (99.9%, 99.9%) |
| 17 | 97.9% (97.9%, 97.9%) | 18.6% (18.6%, 18.6%) | 1.6% (1.6%, 1.6%) | 99.8% (99.8%, 99.9%) |
| 18 | 97.7% (97.7%, 97.7%) | 19.7% (19.7%, 19.7%) | 1.6% (1.6%, 1.6%) | 99.8% (99.8%, 99.8%) |
| 19 | 97.5% (97.5%, 97.5%) | 20.8% (20.8%, 20.8%) | 1.6% (1.6%, 1.6%) | 99.8% (99.8%, 99.8%) |
| 20 | 97.4% (97.4%, 97.4%) | 21.4% (21.4%, 21.4%) | 1.6% (1.6%, 1.6%) | 99.8% (99.8%, 99.8%) |
| 21 | 97.2% (97.2%, 97.2%) | 22.5% (22.5%, 22.5%) | 1.6% (1.6%, 1.6%) | 99.8% (99.8%, 99.8%) |
| 22 | 97.0% (97.0%, 97.0%) | 23.5% (23.5%, 23.5%) | 1.7% (1.7%, 1.7%) | 99.8% (99.8%, 99.8%) |
| 23 | 96.8% (96.8%, 96.8%) | 24.6% (24.5%, 24.6%) | 1.7% (1.7%, 1.7%) | 99.8% (99.8%, 99.8%) |
| 24 | 96.6% (96.6%, 96.6%) | 25.6% (25.5%, 25.6%) | 1.7% (1.7%, 1.7%) | 99.8% (99.8%, 99.8%) |

| | | | | |
|---|---|---|---|---|
| 25 | 96.4% (96.4%, 96.4%) | 26.5% (26.5%, 26.5%) | 1.7% (1.7%, 1.7%) | 99.8% (99.8%, 99.8%) |
| 26 | 96.1% (96.1%, 96.1%) | 28.0% (28.0%, 28.0%) | 1.7% (1.7%, 1.7%) | 99.8% (99.8%, 99.8%) |
| 27 | 95.9% (95.8%, 95.9%) | 28.9% (28.9%, 28.9%) | 1.8% (1.8%, 1.8%) | 99.8% (99.8%, 99.8%) |
| 28 | 95.7% (95.6%, 95.7%) | 29.8% (29.8%, 29.8%) | 1.8% (1.8%, 1.8%) | 99.8% (99.8%, 99.8%) |
| 29 | 95.5% (95.4%, 95.5%) | 30.7% (30.7%, 30.7%) | 1.8% (1.8%, 1.8%) | 99.8% (99.8%, 99.8%) |
| 30 | 95.2% (95.1%, 95.2%) | 31.9% (31.9%, 31.9%) | 1.8% (1.8%, 1.8%) | 99.8% (99.8%, 99.8%) |
| 31 | 94.9% (94.9%, 95.0%) | 32.8% (32.8%, 32.8%) | 1.8% (1.8%, 1.8%) | 99.8% (99.8%, 99.8%) |
| 32 | 94.6% (94.6%, 94.7%) | 34.0% (34.0%, 34.0%) | 1.9% (1.9%, 1.9%) | 99.8% (99.8%, 99.8%) |
| 33 | 94.4% (94.4%, 94.5%) | 34.7% (34.7%, 34.8%) | 1.9% (1.9%, 1.9%) | 99.8% (99.8%, 99.8%) |
| 34 | 94.1% (94.1%, 94.2%) | 35.9% (35.9%, 35.9%) | 1.9% (1.9%, 1.9%) | 99.8% (99.8%, 99.8%) |
| 35 | 93.8% (93.8%, 93.9%) | 37.0% (37.0%, 37.0%) | 1.9% (1.9%, 1.9%) | 99.8% (99.8%, 99.8%) |
| 36 | 93.6% (93.6%, 93.7%) | 37.7% (37.7%, 37.7%) | 2.0% (2.0%, 2.0%) | 99.8% (99.8%, 99.8%) |
| 37 | 93.3% (93.3%, 93.4%) | 38.7% (38.7%, 38.7%) | 2.0% (2.0%, 2.0%) | 99.8% (99.8%, 99.8%) |
| 38 | 93.0% (93.0%, 93.1%) | 39.7% (39.7%, 39.7%) | 2.0% (2.0%, 2.0%) | 99.8% (99.8%, 99.8%) |
| 39 | 92.7% (92.7%, 92.8%) | 40.7% (40.7%, 40.7%) | 2.0% (2.0%, 2.0%) | 99.8% (99.8%, 99.8%) |
| 40 | 92.4% (92.3%, 92.4%) | 41.9% (41.9%, 41.9%) | 2.1% (2.1%, 2.1%) | 99.8% (99.8%, 99.8%) |
| 41 | 92.1% (92.0%, 92.1%) | 42.8% (42.8%, 42.8%) | 2.1% (2.1%, 2.1%) | 99.8% (99.8%, 99.8%) |
| 42 | 91.8% (91.8%, 91.8%) | 43.7% (43.7%, 43.7%) | 2.1% (2.1%, 2.1%) | 99.8% (99.7%, 99.8%) |
| 43 | 91.4% (91.4%, 91.5%) | 44.8% (44.8%, 44.8%) | 2.2% (2.2%, 2.2%) | 99.7% (99.7%, 99.7%) |
| 44 | 91.0% (91.0%, 91.1%) | 45.9% (45.9%, 45.9%) | 2.2% (2.2%, 2.2%) | 99.7% (99.7%, 99.7%) |
| 45 | 90.7% (90.6%, 90.7%) | 46.9% (46.9%, 46.9%) | 2.2% (2.2%, 2.2%) | 99.7% (99.7%, 99.7%) |
| 46 | 90.4% (90.4%, 90.5%) | 47.7% (47.7%, 47.7%) | 2.2% (2.2%, 2.3%) | 99.7% (99.7%, 99.7%) |
| 47 | 90.0% (90.0%, 90.0%) | 48.9% (48.9%, 48.9%) | 2.3% (2.3%, 2.3%) | 99.7% (99.7%, 99.7%) |
| 48 | 89.6% (89.6%, 89.7%) | 49.8% (49.8%, 49.8%) | 2.3% (2.3%, 2.3%) | 99.7% (99.7%, 99.7%) |
| 49 | 89.3% (89.3%, 89.3%) | 50.7% (50.7%, 50.7%) | 2.4% (2.3%, 2.4%) | 99.7% (99.7%, 99.7%) |
| 50 | 88.9% (88.8%, 88.9%) | 51.8% (51.8%, 51.8%) | 2.4% (2.4%, 2.4%) | 99.7% (99.7%, 99.7%) |
| 51 | 88.5% (88.4%, 88.5%) | 52.8% (52.8%, 52.8%) | 2.4% (2.4%, 2.4%) | 99.7% (99.7%, 99.7%) |
| 52 | 88.1% (88.0%, 88.1%) | 53.8% (53.8%, 53.8%) | 2.5% (2.5%, 2.5%) | 99.7% (99.7%, 99.7%) |
| 53 | 87.7% (87.6%, 87.7%) | 54.7% (54.7%, 54.7%) | 2.5% (2.5%, 2.5%) | 99.7% (99.7%, 99.7%) |
| 54 | 87.3% (87.2%, 87.3%) | 55.6% (55.6%, 55.6%) | 2.5% (2.5%, 2.6%) | 99.7% (99.7%, 99.7%) |

| | | | | |
|---|---|---|---|---|
| 55 | 86.8% (86.8%, 86.9%) | 56.7% (56.7%, 56.7%) | 2.6% (2.6%, 2.6%) | 99.7% (99.7%, 99.7%) |
| 56 | 86.4% (86.3%, 86.4%) | 57.7% (57.7%, 57.7%) | 2.6% (2.6%, 2.6%) | 99.7% (99.7%, 99.7%) |
| 57 | 85.9% (85.9%, 86.0%) | 58.6% (58.6%, 58.6%) | 2.7% (2.7%, 2.7%) | 99.7% (99.7%, 99.7%) |
| 58 | 85.4% (85.4%, 85.5%) | 59.7% (59.7%, 59.7%) | 2.7% (2.7%, 2.7%) | 99.7% (99.7%, 99.7%) |
| 59 | 85.0% (84.9%, 85.0%) | 60.5% (60.5%, 60.5%) | 2.8% (2.8%, 2.8%) | 99.7% (99.7%, 99.7%) |
| 60 | 84.5% (84.4%, 84.5%) | 61.5% (61.5%, 61.5%) | 2.8% (2.8%, 2.8%) | 99.7% (99.7%, 99.7%) |
| 61 | 83.9% (83.9%, 84.0%) | 62.5% (62.5%, 62.6%) | 2.9% (2.9%, 2.9%) | 99.7% (99.7%, 99.7%) |
| 62 | 83.4% (83.3%, 83.5%) | 63.5% (63.5%, 63.6%) | 3.0% (2.9%, 3.0%) | 99.7% (99.7%, 99.7%) |
| 63 | 82.9% (82.8%, 82.9%) | 64.5% (64.5%, 64.5%) | 3.0% (3.0%, 3.0%) | 99.6% (99.6%, 99.6%) |
| 64 | 82.3% (82.2%, 82.4%) | 65.5% (65.5%, 65.5%) | 3.1% (3.1%, 3.1%) | 99.6% (99.6%, 99.6%) |
| 65 | 81.7% (81.7%, 81.8%) | 66.4% (66.4%, 66.5%) | 3.1% (3.1%, 3.1%) | 99.6% (99.6%, 99.6%) |
| 66 | 81.2% (81.1%, 81.2%) | 67.4% (67.3%, 67.4%) | 3.2% (3.2%, 3.2%) | 99.6% (99.6%, 99.6%) |
| 67 | 80.6% (80.5%, 80.6%) | 68.3% (68.3%, 68.3%) | 3.3% (3.3%, 3.3%) | 99.6% (99.6%, 99.6%) |
| 68 | 80.0% (79.9%, 80.0%) | 69.3% (69.3%, 69.3%) | 3.3% (3.3%, 3.4%) | 99.6% (99.6%, 99.6%) |
| 69 | 79.3% (79.2%, 79.3%) | 70.3% (70.3%, 70.3%) | 3.4% (3.4%, 3.4%) | 99.6% (99.6%, 99.6%) |
| 70 | 78.6% (78.6%, 78.7%) | 71.2% (71.2%, 71.3%) | 3.5% (3.5%, 3.5%) | 99.6% (99.6%, 99.6%) |
| 71 | 78.0% (77.9%, 78.0%) | 72.2% (72.2%, 72.2%) | 3.6% (3.6%, 3.6%) | 99.6% (99.6%, 99.6%) |
| 72 | 77.3% (77.2%, 77.3%) | 73.2% (73.2%, 73.2%) | 3.7% (3.7%, 3.7%) | 99.6% (99.6%, 99.6%) |
| 73 | 76.6% (76.5%, 76.6%) | 74.1% (74.1%, 74.1%) | 3.8% (3.8%, 3.8%) | 99.6% (99.6%, 99.6%) |
| 74 | 75.8% (75.8%, 75.9%) | 75.1% (75.0%, 75.1%) | 3.9% (3.9%, 3.9%) | 99.6% (99.6%, 99.6%) |
| 75 | 75.1% (75.0%, 75.1%) | 76.0% (76.0%, 76.0%) | 4.0% (4.0%, 4.0%) | 99.6% (99.6%, 99.6%) |
| 76 | 74.2% (74.2%, 74.3%) | 77.0% (77.0%, 77.0%) | 4.1% (4.1%, 4.1%) | 99.6% (99.6%, 99.6%) |
| 77 | 73.4% (73.4%, 73.5%) | 77.9% (77.9%, 77.9%) | 4.2% (4.2%, 4.2%) | 99.5% (99.5%, 99.6%) |
| 78 | 72.6% (72.5%, 72.6%) | 78.9% (78.9%, 78.9%) | 4.4% (4.4%, 4.4%) | 99.5% (99.5%, 99.5%) |
| 79 | 71.7% (71.6%, 71.7%) | 79.8% (79.8%, 79.8%) | 4.5% (4.5%, 4.5%) | 99.5% (99.5%, 99.5%) |
| 80 | 70.7% (70.7%, 70.8%) | 80.8% (80.8%, 80.8%) | 4.7% (4.7%, 4.7%) | 99.5% (99.5%, 99.5%) |
| 81 | 69.7% (69.7%, 69.8%) | 81.7% (81.7%, 81.7%) | 4.8% (4.8%, 4.8%) | 99.5% (99.5%, 99.5%) |
| 82 | 68.7% (68.6%, 68.8%) | 82.7% (82.7%, 82.7%) | 5.0% (5.0%, 5.0%) | 99.5% (99.5%, 99.5%) |
| 83 | 67.6% (67.6%, 67.7%) | 83.6% (83.6%, 83.6%) | 5.2% (5.2%, 5.2%) | 99.5% (99.5%, 99.5%) |
| 84 | 66.5% (66.4%, 66.6%) | 84.6% (84.6%, 84.6%) | 5.4% (5.4%, 5.4%) | 99.5% (99.5%, 99.5%) |

| | | | | |
|---|---|---|---|---|
| 85 | 65.3% (65.2%, 65.3%) | 85.5% (85.5%, 85.5%) | 5.7% (5.6%, 5.7%) | 99.5% (99.5%, 99.5%) |
| 86 | 64.0% (63.9%, 64.1%) | 86.5% (86.5%, 86.5%) | 5.9% (5.9%, 5.9%) | 99.4% (99.4%, 99.5%) |
| 87 | 62.6% (62.6%, 62.7%) | 87.4% (87.4%, 87.4%) | 6.2% (6.2%, 6.2%) | 99.4% (99.4%, 99.4%) |
| 88 | 61.2% (61.1%, 61.2%) | 88.4% (88.4%, 88.4%) | 6.5% (6.5%, 6.5%) | 99.4% (99.4%, 99.4%) |
| 89 | 59.6% (59.5%, 59.7%) | 89.3% (89.3%, 89.3%) | 6.9% (6.9%, 6.9%) | 99.4% (99.4%, 99.4%) |
| 90 | 57.9% (57.9%, 58.0%) | 90.2% (90.2%, 90.2%) | 7.3% (7.3%, 7.3%) | 99.4% (99.4%, 99.4%) |
| 91 | 56.1% (56.0%, 56.2%) | 91.2% (91.2%, 91.2%) | 7.8% (7.8%, 7.8%) | 99.4% (99.4%, 99.4%) |
| 92 | 54.0% (54.0%, 54.1%) | 92.1% (92.1%, 92.1%) | 8.4% (8.3%, 8.4%) | 99.3% (99.3%, 99.3%) |
| 93 | 51.8% (51.7%, 51.8%) | 93.1% (93.1%, 93.1%) | 9.0% (9.0%, 9.1%) | 99.3% (99.3%, 99.3%) |
| 94 | 49.2% (49.1%, 49.3%) | 94.0% (94.0%, 94.0%) | 9.9% (9.8%, 9.9%) | 99.3% (99.3%, 99.3%) |
| 95 | 46.3% (46.2%, 46.4%) | 95.0% (95.0%, 95.0%) | 10.9% (10.9%, 10.9%) | 99.3% (99.3%, 99.3%) |
| 96 | 42.9% (42.8%, 42.9%) | 95.9% (95.9%, 95.9%) | 12.3% (12.2%, 12.3%) | 99.2% (99.2%, 99.2%) |
| 97 | 38.6% (38.5%, 38.7%) | 96.9% (96.9%, 96.9%) | 14.2% (14.1%, 14.2%) | 99.2% (99.2%, 99.2%) |
| 98 | 33.1% (33.0%, 33.2%) | 97.9% (97.9%, 97.9%) | 17.1% (17.0%, 17.1%) | 99.1% (99.1%, 99.1%) |
| 99 | 24.6% (24.5%, 24.7%) | 98.9% (98.9%, 98.9%) | 22.4% (22.3%, 22.4%) | 99.0% (99.0%, 99.0%) |
| 99.1 | 23.4% (23.4%, 23.5%) | 99.0% (99.0%, 99.0%) | 23.2% (23.1%, 23.3%) | 99.0% (99.0%, 99.0%) |
| 99.2 | 22.2% (22.1%, 22.3%) | 99.1% (99.1%, 99.1%) | 24.1% (24.1%, 24.2%) | 99.0% (99.0%, 99.0%) |
| 99.3 | 20.8% (20.8%, 20.9%) | 99.2% (99.2%, 99.2%) | 25.2% (25.1%, 25.3%) | 98.9% (98.9%, 99.0%) |
| 99.4 | 19.3% (19.2%, 19.3%) | 99.3% (99.3%, 99.3%) | 26.4% (26.3%, 26.5%) | 98.9% (98.9%, 98.9%) |
| 99.5 | 17.6% (17.5%, 17.6%) | 99.4% (99.4%, 99.4%) | 27.8% (27.8%, 27.9%) | 98.9% (98.9%, 98.9%) |
| 99.6 | 15.6% (15.5%, 15.7%) | 99.5% (99.5%, 99.5%) | 29.6% (29.5%, 29.7%) | 98.9% (98.9%, 98.9%) |
| 99.7 | 13.3% (13.3%, 13.4%) | 99.6% (99.6%, 99.6%) | 31.8% (31.7%, 31.9%) | 98.9% (98.9%, 98.9%) |
| 99.8 | 10.4% (10.4%, 10.5%) | 99.7% (99.7%, 99.7%) | 34.9% (34.8%, 35.0%) | 98.8% (98.8%, 98.8%) |
| 99.9 | 6.7% (6.7%, 6.8%) | 99.9% (99.9%, 99.9%) | 40.2% (40.0%, 40.4%) | 98.8% (98.8%, 98.8%) |
| 99.91 | 6.3% (6.2%, 6.3%) | 99.9% (99.9%, 99.9%) | 41.0% (40.8%, 41.2%) | 98.8% (98.8%, 98.8%) |
| 99.92 | 5.8% (5.8%, 5.8%) | 99.9% (99.9%, 99.9%) | 41.9% (41.7%, 42.1%) | 98.8% (98.8%, 98.8%) |
| 99.93 | 5.3% (5.3%, 5.3%) | 99.9% (99.9%, 99.9%) | 42.9% (42.7%, 43.1%) | 98.8% (98.8%, 98.8%) |
| 99.94 | 4.8% (4.7%, 4.8%) | 99.9% (99.9%, 99.9%) | 44.0% (43.8%, 44.3%) | 98.7% (98.7%, 98.8%) |
| 99.95 | 4.2% (4.2%, 4.2%) | 99.9% (99.9%, 99.9%) | 45.4% (45.2%, 45.6%) | 98.7% (98.7%, 98.7%) |
| 99.96 | 3.6% (3.5%, 3.6%) | 99.9% (99.9%, 99.9%) | 47.0% (46.7%, 47.3%) | 98.7% (98.7%, 98.7%) |

| | | | | |
|---|---|---|---|---|
| 99.97 | 2.9% (2.8%, 2.9%) | 100.0% (100.0%, 100.0%) | 49.1% (48.7%, 49.4%) | 98.7% (98.7%, 98.7%) |
| 99.98 | 2.1% (2.1%, 2.1%) | 100.0% (100.0%, 100.0%) | 51.8% (51.4%, 52.2%) | 98.7% (98.7%, 98.7%) |
| 99.99 | 1.2% (1.2%, 1.3%) | 100.0% (100.0%, 100.0%) | 56.3% (55.8%, 56.8%) | 98.7% (98.7%, 98.7%) |
| 100 | 0.7% (0.7%, 0.7%) | 100.0% (100.0%, 100.0%) | 61.0% (60.4%, 61.7%) | 98.7% (98.7%, 98.7%) |

Abbreviations: UW = UW Health University Hospital; YNHHS = Yale New Haven Health System

**Table E10.** Full retrospective cohort eCARTv2 test characteristics for the primary outcome of deterioration (N=1,769,461 encounters, n=132,873,833 observations).

| eCARTv2 | Sensitivity | Specificity | Positive Predictive Value | Negative Predictive Value |
|---|---|---|---|---|
| | | | | |
| 0 | 100.0% (100.0%, 100.0%) | 0.0% (0.0%, 0.0%) | 1.3% (1.3%, 1.3%) | N/A |
| 5 | 99.4% (99.4%, 99.4%) | 4.1% (4.1%, 4.1%) | 1.4% (1.4%, 1.4%) | 99.8% (99.8%, 99.8%) |
| 16 | 96.7% (96.7%, 96.7%) | 15.8% (15.8%, 15.8%) | 1.5% (1.5%, 1.5%) | 99.7% (99.7%, 99.7%) |
| 30 | 91.9% (91.8%, 91.9%) | 30.8% (30.8%, 30.8%) | 1.7% (1.7%, 1.7%) | 99.7% (99.6%, 99.7%) |
| 43 | 86.1% (86.0%, 86.1%) | 44.7% (44.7%, 44.7%) | 2.0% (2.0%, 2.0%) | 99.6% (99.6%, 99.6%) |
| 53 | 80.3% (80.2%, 80.3%) | 55.9% (55.9%, 55.9%) | 2.4% (2.4%, 2.4%) | 99.5% (99.5%, 99.5%) |
| 61 | 74.9% (74.8%, 75.0%) | 64.4% (64.4%, 64.4%) | 2.7% (2.7%, 2.7%) | 99.5% (99.5%, 99.5%) |
| 68 | 70.1% (70.0%, 70.2%) | 70.9% (70.9%, 70.9%) | 3.1% (3.1%, 3.1%) | 99.4% (99.4%, 99.4%) |
| 73 | 65.8% (65.8%, 65.9%) | 75.9% (75.9%, 75.9%) | 3.5% (3.5%, 3.5%) | 99.4% (99.4%, 99.4%) |
| 76 | 61.9% (61.9%, 62.0%) | 79.8% (79.8%, 79.8%) | 3.9% (3.9%, 3.9%) | 99.4% (99.4%, 99.4%) |
| 80 | 58.5% (58.4%, 58.5%) | 82.8% (82.8%, 82.8%) | 4.3% (4.3%, 4.3%) | 99.3% (99.3%, 99.3%) |
| 82 | 55.4% (55.3%, 55.4%) | 85.2% (85.2%, 85.2%) | 4.7% (4.7%, 4.7%) | 99.3% (99.3%, 99.3%) |
| 84 | 52.6% (52.5%, 52.7%) | 87.1% (87.1%, 87.1%) | 5.2% (5.1%, 5.2%) | 99.3% (99.3%, 99.3%) |
| 86 | 50.1% (50.0%, 50.1%) | 88.7% (88.7%, 88.7%) | 5.6% (5.6%, 5.6%) | 99.3% (99.3%, 99.3%) |
| 87 | 47.7% (47.7%, 47.8%) | 90.0% (90.0%, 90.0%) | 6.0% (6.0%, 6.0%) | 99.2% (99.2%, 99.2%) |
| 89 | 45.6% (45.6%, 45.7%) | 91.1% (91.1%, 91.1%) | 6.4% (6.4%, 6.4%) | 99.2% (99.2%, 99.2%) |
| 90 | 43.7% (43.6%, 43.8%) | 92.0% (92.0%, 92.0%) | 6.8% (6.7%, 6.8%) | 99.2% (99.2%, 99.2%) |
| 91 | 40.3% (40.3%, 40.4%) | 93.4% (93.4%, 93.4%) | 7.5% (7.5%, 7.5%) | 99.2% (99.2%, 99.2%) |
| 92 | 38.8% (38.8%, 38.9%) | 94.0% (94.0%, 94.0%) | 7.9% (7.9%, 7.9%) | 99.1% (99.1%, 99.1%) |
| 93 | 37.4% (37.3%, 37.5%) | 94.5% (94.4%, 94.5%) | 8.2% (8.2%, 8.3%) | 99.1% (99.1%, 99.1%) |
| 94 | 34.9% (34.8%, 35.0%) | 95.3% (95.3%, 95.3%) | 8.9% (8.9%, 8.9%) | 99.1% (99.1%, 99.1%) |
| 95 | 31.6% (31.6%, 31.7%) | 96.1% (96.1%, 96.2%) | 9.9% (9.8%, 9.9%) | 99.1% (99.1%, 99.1%) |
| 96 | 28.1% (28.0%, 28.2%) | 97.0% (97.0%, 97.0%) | 11.0% (10.9%, 11.0%) | 99.0% (99.0%, 99.0%) |
| 97 | 24.7% (24.7%, 24.8%) | 97.6% (97.6%, 97.6%) | 12.2% (12.2%, 12.3%) | 99.0% (99.0%, 99.0%) |

| eCARTv2 | Sensitivity | Specificity | Positive Predictive Value | Negative Predictive Value |
|---|---|---|---|---|
| 98 | 20.0% (19.9%, 20.0%) | 98.4% (98.4%, 98.4%) | 14.2% (14.2%, 14.2%) | 98.9% (98.9%, 98.9%) |
| 99 | 13.4% (13.3%, 13.4%) | 99.1% (99.1%, 99.1%) | 17.0% (16.9%, 17.1%) | 98.9% (98.8%, 98.9%) |
| 99.1 | 12.5% (12.5%, 12.6%) | 99.2% (99.2%, 99.2%) | 17.3% (17.3%, 17.4%) | 98.8% (98.8%, 98.8%) |
| 99.2 | 11.7% (11.6%, 11.7%) | 99.3% (99.3%, 99.3%) | 17.6% (17.6%, 17.7%) | 98.8% (98.8%, 98.8%) |
| 99.3 | 10.6% (10.6%, 10.7%) | 99.4% (99.4%, 99.4%) | 17.9% (17.8%, 18.0%) | 98.8% (98.8%, 98.8%) |
| 99.4 | 9.5% (9.5%, 9.6%) | 99.4% (99.4%, 99.4%) | 18.1% (18.0%, 18.2%) | 98.8% (98.8%, 98.8%) |
| 99.5 | 8.4% (8.4%, 8.5%) | 99.5% (99.5%, 99.5%) | 18.3% (18.2%, 18.4%) | 98.8% (98.8%, 98.8%) |
| 99.6 | 7.2% (7.2%, 7.2%) | 99.6% (99.6%, 99.6%) | 18.2% (18.1%, 18.3%) | 98.8% (98.8%, 98.8%) |
| 99.7 | 5.8% (5.8%, 5.9%) | 99.6% (99.6%, 99.6%) | 17.8% (17.7%, 17.9%) | 98.8% (98.8%, 98.8%) |
| 99.8 | 4.4% (4.3%, 4.4%) | 99.7% (99.7%, 99.7%) | 16.8% (16.7%, 16.9%) | 98.7% (98.7%, 98.7%) |
| 99.9 | 2.6% (2.6%, 2.6%) | 99.8% (99.8%, 99.8%) | 13.8% (13.7%, 13.9%) | 98.7% (98.7%, 98.7%) |
| 99.91 | 2.4% (2.4%, 2.4%) | 99.8% (99.8%, 99.8%) | 13.2% (13.1%, 13.3%) | 98.7% (98.7%, 98.7%) |
| 99.92 | 2.2% (2.2%, 2.2%) | 99.8% (99.8%, 99.8%) | 12.7% (12.6%, 12.8%) | 98.7% (98.7%, 98.7%) |
| 99.93 | 2.0% (2.0%, 2.1%) | 99.8% (99.8%, 99.8%) | 12.2% (12.0%, 12.3%) | 98.7% (98.7%, 98.7%) |
| 99.94 | 1.8% (1.8%, 1.8%) | 99.8% (99.8%, 99.8%) | 11.3% (11.1%, 11.4%) | 98.7% (98.7%, 98.7%) |
| 99.95 | 1.6% (1.5%, 1.6%) | 99.8% (99.8%, 99.8%) | 10.5% (10.3%, 10.6%) | 98.7% (98.7%, 98.7%) |
| 99.96 | 1.3% (1.3%, 1.3%) | 99.8% (99.8%, 99.8%) | 9.3% (9.2%, 9.4%) | 98.7% (98.7%, 98.7%) |
| 99.97 | 1.1% (1.1%, 1.1%) | 99.8% (99.8%, 99.8%) | 8.1% (8.0%, 8.2%) | 98.7% (98.7%, 98.7%) |
| 99.98 | 0.8% (0.7%, 0.8%) | 99.9% (99.9%, 99.9%) | 6.4% (6.2%, 6.5%) | 98.7% (98.7%, 98.7%) |
| 99.99 | 0.5% (0.5%, 0.5%) | 99.9% (99.9%, 99.9%) | 4.4% (4.3%, 4.5%) | 98.7% (98.7%, 98.7%) |
| 100 | 0.0% (0.0%, 0.0%) | 99.9% (99.9%, 99.9%) | 0.5% (0.5%, 0.5%) | 98.7% (98.7%, 98.7%) |

**Table E11.** Full retrospective cohort NEWS test characteristics for the primary outcome of deterioration (N=1,769,461 encounters, n=132,873,833 observations).

| NEWS | Sensitivity | Specificity | Positive Predictive Value | Negative Predictive Value |
|---|---|---|---|---|
| 0 | 100.0% (100.0%, 100.0%) | 0.0% (0.0%, 0.0%) | 1.3% (1.3%, 1.3%) | N/A |
| 1 | 90.8% (90.8%, 90.9%) | 29.7% (29.7%, 29.7%) | 1.7% (1.7%, 1.7%) | 99.6% (99.6%, 99.6%) |
| 2 | 83.4% (83.3%, 83.4%) | 50.5% (50.5%, 50.5%) | 2.2% (2.2%, 2.2%) | 99.6% (99.6%, 99.6%) |
| 3 | 72.3% (72.2%, 72.4%) | 68.2% (68.2%, 68.2%) | 2.9% (2.9%, 2.9%) | 99.5% (99.5%, 99.5%) |
| 4 | 60.2% (60.1%, 60.3%) | 80.8% (80.8%, 80.8%) | 4.0% (4.0%, 4.0%) | 99.3% (99.3%, 99.4%) |
| 5 | 49.7% (49.6%, 49.8%) | 88.3% (88.3%, 88.3%) | 5.4% (5.3%, 5.4%) | 99.2% (99.2%, 99.3%) |
| 6 | 37.9% (37.8%, 37.9%) | 93.6% (93.6%, 93.6%) | 7.3% (7.3%, 7.3%) | 99.1% (99.1%, 99.1%) |
| 7 | 28.0% (27.9%, 28.1%) | 96.6% (96.6%, 96.6%) | 9.9% (9.8%, 9.9%) | 99.0% (99.0%, 99.0%) |
| 8 | 20.1% (20.1%, 20.2%) | 98.2% (98.2%, 98.2%) | 13.0% (12.9%, 13.0%) | 98.9% (98.9%, 98.9%) |
| 9 | 13.3% (13.3%, 13.4%) | 99.1% (99.1%, 99.1%) | 16.8% (16.7%, 16.8%) | 98.9% (98.8%, 98.9%) |
| 10 | 8.6% (8.5%, 8.6%) | 99.6% (99.6%, 99.6%) | 21.4% (21.3%, 21.5%) | 98.8% (98.8%, 98.8%) |
| 11 | 5.2% (5.2%, 5.3%) | 99.8% (99.8%, 99.8%) | 26.5% (26.4%, 26.6%) | 98.8% (98.8%, 98.8%) |
| 12 | 3.0% (2.9%, 3.0%) | 99.9% (99.9%, 99.9%) | 31.9% (31.7%, 32.2%) | 98.7% (98.7%, 98.7%) |
| 13 | 1.6% (1.6%, 1.6%) | 100.0% (100.0%, 100.0%) | 37.8% (37.4%, 38.2%) | 98.7% (98.7%, 98.7%) |
| 14 | 0.8% (0.8%, 0.8%) | 100.0% (100.0%, 100.0%) | 44.0% (43.4%, 44.5%) | 98.7% (98.7%, 98.7%) |
| 15 | 0.4% (0.3%, 0.4%) | 100.0% (100.0%, 100.0%) | 49.6% (48.7%, 50.4%) | 98.7% (98.7%, 98.7%) |
| 16 | 0.1% (0.1%, 0.2%) | 100.0% (100.0%, 100.0%) | 57.5% (56.0%, 58.9%) | 98.7% (98.7%, 98.7%) |
| 17 | 0.1% (0.1%, 0.1%) | 100.0% (100.0%, 100.0%) | 66.4% (63.9%, 68.8%) | 98.7% (98.7%, 98.7%) |
| 18 | 0.0% (0.0%, 0.0%) | 100.0% (100.0%, 100.0%) | 70.8% (65.6%, 75.6%) | 98.7% (98.7%, 98.7%) |
| 19 | 0.0% (0.0%, 0.0%) | 100.0% (100.0%, 100.0%) | 82.4% (71.2%, 90.5%) | 98.7% (98.7%, 98.7%) |
| 20 | 0.0% (0.0%, 0.0%) | 100.0% (100.0%, 100.0%) | 89.3% (71.8%, 97.7%) | 98.7% (98.7%, 98.7%) |

Abbreviations: UW = UW Health University Hospital; YNHHS = Yale New Haven Health System; NEWS = National Early Warning Score

**Table E12.** Full retrospective cohort MEWS test characteristics for the primary outcome of deterioration (N=1,769,461 encounters, n=132,873,833 observations).

| MEWS | Sensitivity | Specificity | Positive Predictive Value | Negative Predictive Value |
|---|---|---|---|---|
| 0 | 100.0% (100.0%, 100.0%) | 0.0% (0.0%, 0.0%) | 1.3% (1.3%, 1.3%) | N/A |
| 1 | 97.9% (97.9%, 97.9%) | 3.0% (3.0%, 3.0%) | 1.3% (1.3%, 1.3%) | 99.1% (99.0%, 99.1%) |
| 2 | 62.2% (62.1%, 62.2%) | 72.6% (72.6%, 72.6%) | 2.9% (2.9%, 2.9%) | 99.3% (99.3%, 99.3%) |
| 3 | 38.9% (38.8%, 39.0%) | 91.5% (91.5%, 91.5%) | 5.7% (5.7%, 5.7%) | 99.1% (99.1%, 99.1%) |
| 4 | 22.6% (22.5%, 22.7%) | 97.4% (97.4%, 97.4%) | 10.4% (10.4%, 10.5%) | 99.0% (99.0%, 99.0%) |
| 5 | 12.1% (12.0%, 12.1%) | 99.2% (99.2%, 99.2%) | 16.9% (16.9%, 17.0%) | 98.8% (98.8%, 98.8%) |
| 6 | 6.0% (5.9%, 6.0%) | 99.7% (99.7%, 99.8%) | 24.0% (23.9%, 24.2%) | 98.8% (98.8%, 98.8%) |
| 7 | 2.7% (2.7%, 2.8%) | 99.9% (99.9%, 99.9%) | 31.5% (31.3%, 31.7%) | 98.7% (98.7%, 98.7%) |
| 8 | 1.2% (1.2%, 1.2%) | 100.0% (100.0%, 100.0%) | 38.2% (37.8%, 38.6%) | 98.7% (98.7%, 98.7%) |
| 9 | 0.5% (0.5%, 0.5%) | 100.0% (100.0%, 100.0%) | 45.0% (44.3%, 45.8%) | 98.7% (98.7%, 98.7%) |
| 10 | 0.2% (0.2%, 0.2%) | 100.0% (100.0%, 100.0%) | 51.2% (50.0%, 52.5%) | 98.7% (98.7%, 98.7%) |
| 11 | 0.1% (0.1%, 0.1%) | 100.0% (100.0%, 100.0%) | 60.4% (58.0%, 62.9%) | 98.7% (98.7%, 98.7%) |
| 12 | 0.0% (0.0%, 0.0%) | 100.0% (100.0%, 100.0%) | 69.9% (64.6%, 74.9%) | 98.7% (98.7%, 98.7%) |
| 13 | 0.0% (0.0%, 0.0%) | 100.0% (100.0%, 100.0%) | 79.3% (66.6%, 88.8%) | 98.7% (98.7%, 98.7%) |
| 14 | 0.0% (0.0%, 0.0%) | 100.0% (100.0%, 100.0%) | 79.2% (57.8%, 92.9%) | 98.7% (98.7%, 98.7%) |

Abbreviations: UW = UW Health University Hospital; YNHHS = Yale New Haven Health System; MEWS = Modified Early Warning Score

**Figure E1.** eCARTv5 partial plots. Partial dependence plots of the association between maximum respiratory rate in the prior 24 hours (A), delivered FiO2 (B), minimum systolic blood pressure in the prior 24 hours, and heart rate (D) and the risk of the outcome.

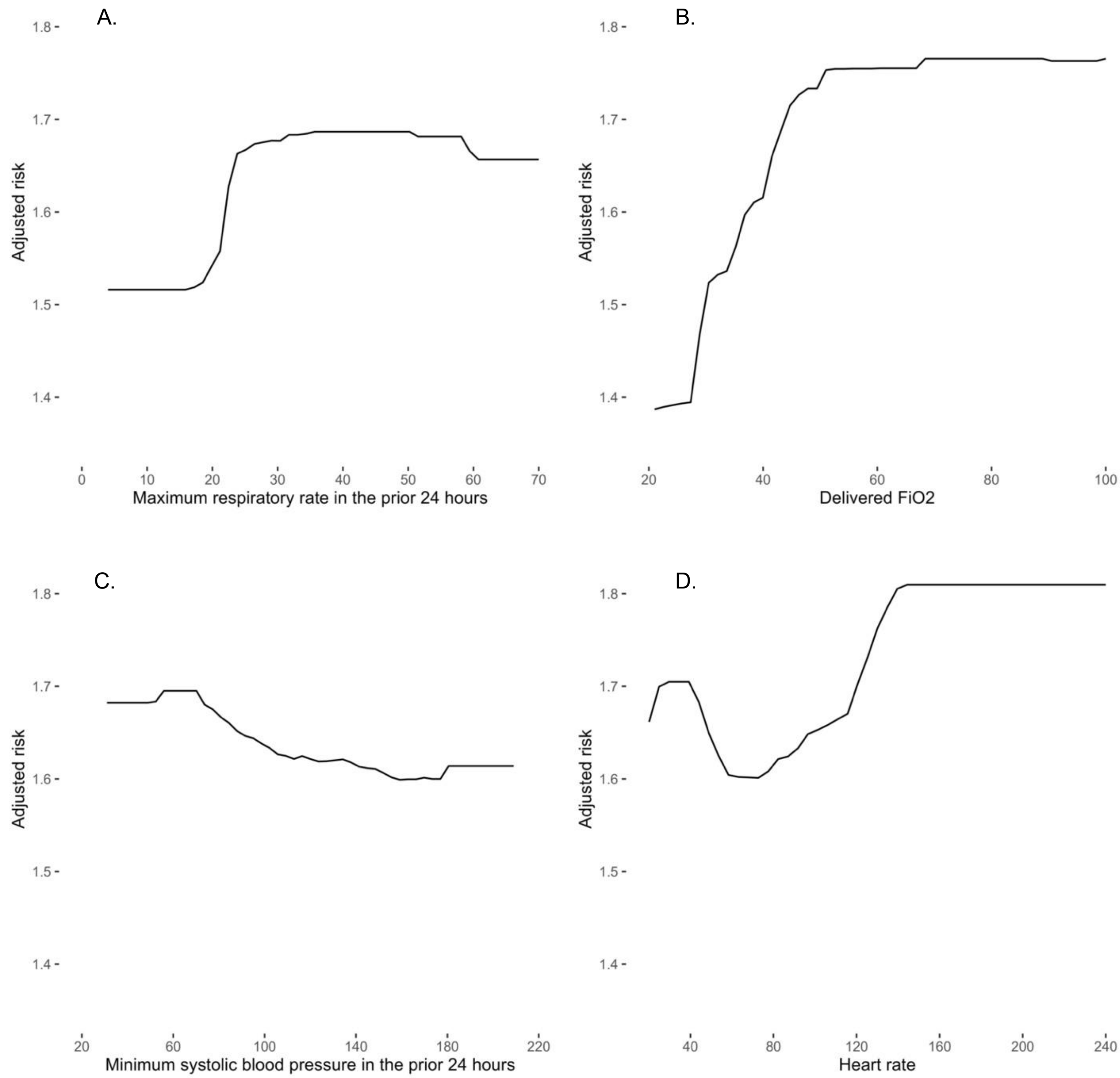


Abbreviations: FiO2 = Fraction of Inspired Oxygen

**Figure E2.** eCARTv5 calibration curve. The figure compares the actual probability (Y-axis) compared to the model's outputted predicted probability (X-axis) for the outcome of deterioration within 24 hours in the prospective test set.

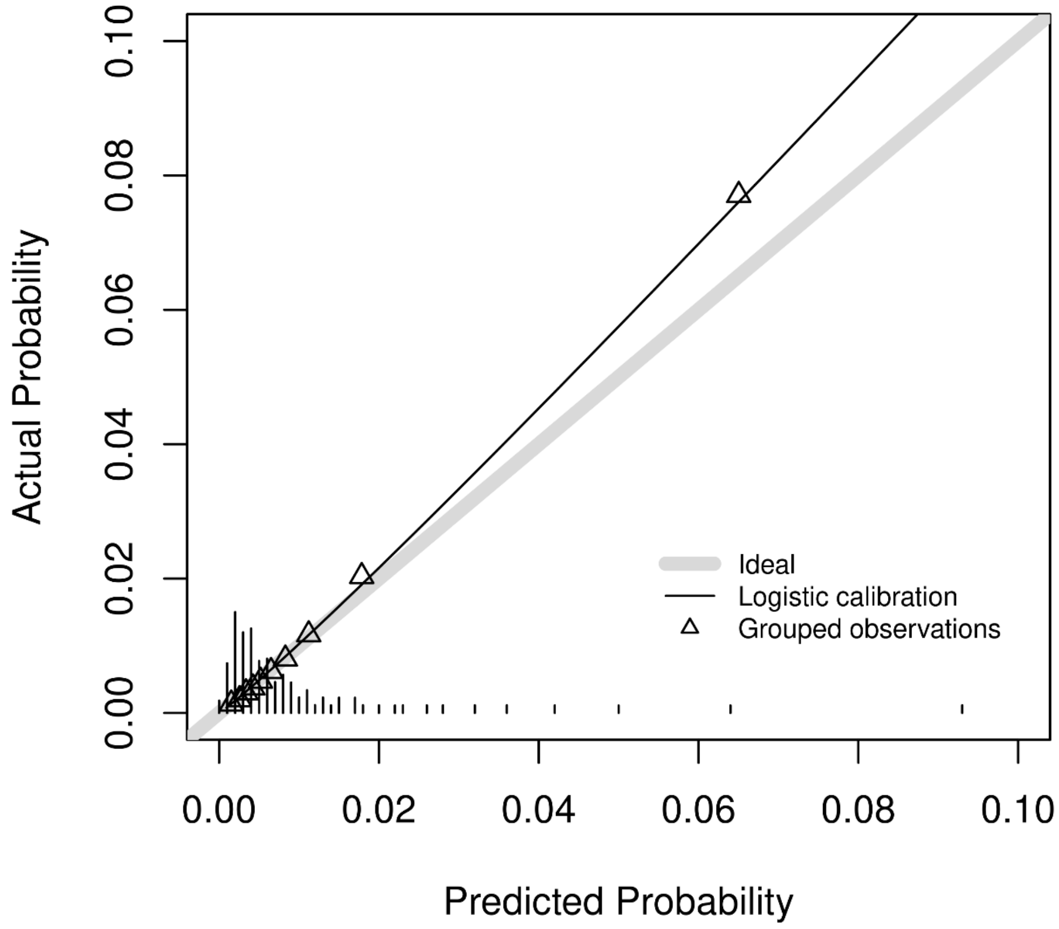

**Figure E3.** Outcome rate for different NEWS values.

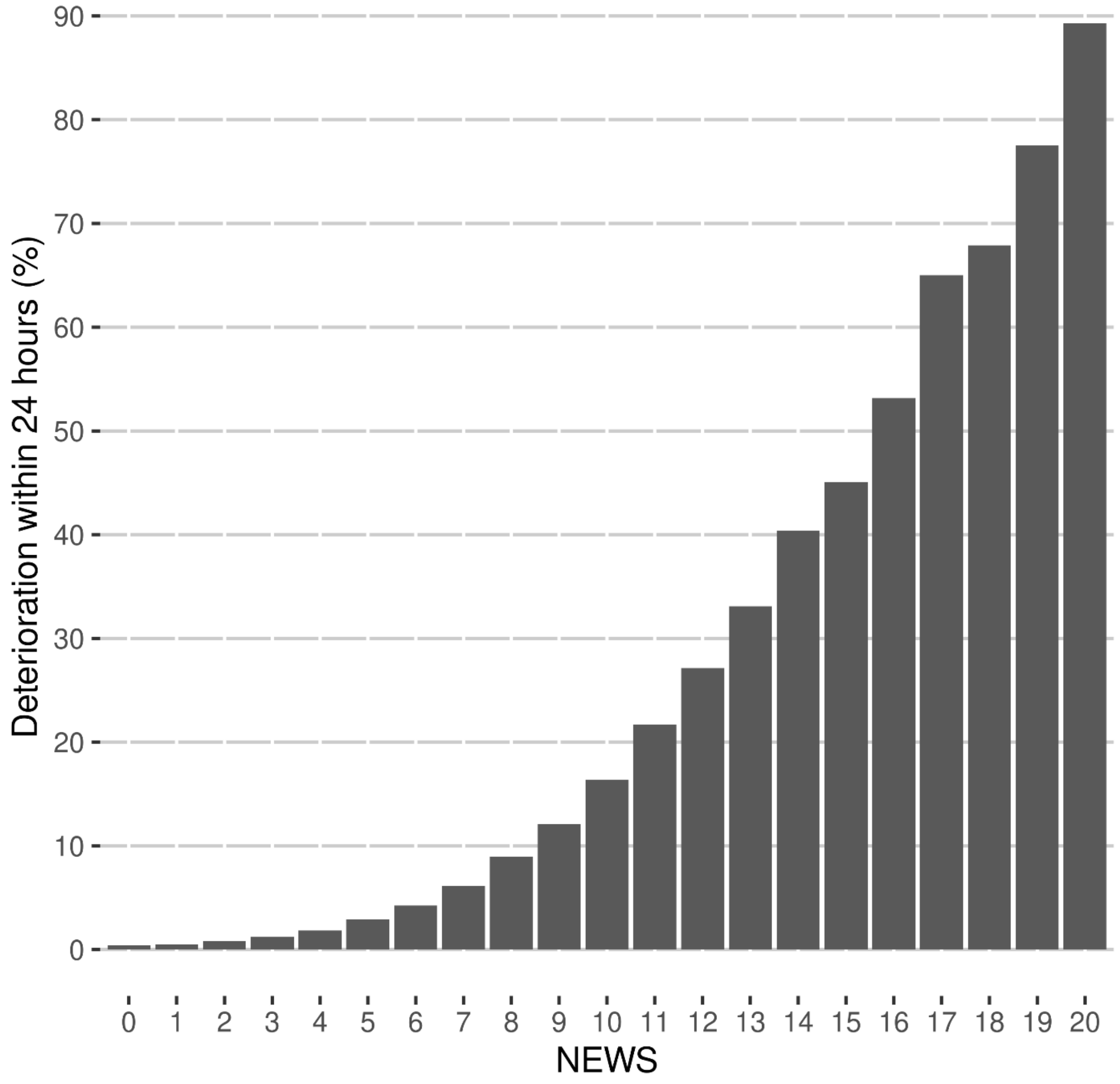

**Figure E4.** Outcome rate for different MEWS values.

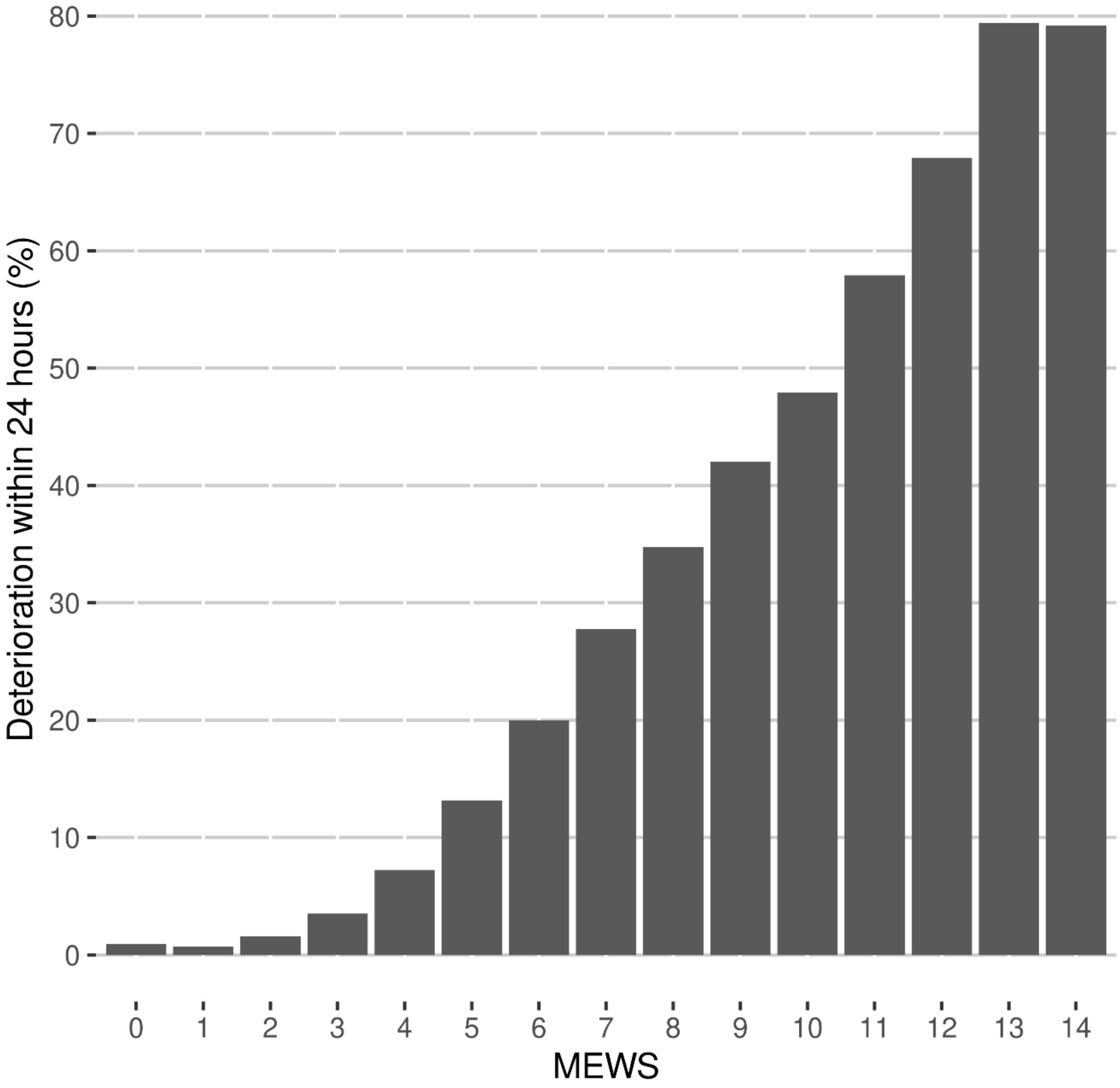

Supplement: Supplementary file 1 [file cc9-7-e1232-s001.pdf]
